# Tau mediates the impact of amyloid and vascular disease burden on the trajectory of clinical symptoms

**DOI:** 10.1002/alz.70831

**Published:** 2025-10-30

**Authors:** Lianlian Du, Rebecca E. Langhough, Bruce P. Hermann, Erin M. Jonaitis, Tobey J. Betthauser, Leonardo A. Rivera‐Rivera, Karly A. Cody, Nathaniel A. Chin, Robert V. Cadman, Kevin M. Johnson, Aaron S. Field, Sanjay Asthana, Laura Eisenmenger, Bradley T. Christian, Sterling C. Johnson

**Affiliations:** ^1^ Wisconsin Alzheimer's Institute University of Wisconsin–Madison School of Medicine and Public Health Madison Wisconsin USA; ^2^ Wisconsin Alzheimer's Disease Research Center, Department of Medicine University of Wisconsin–Madison School of Medicine and Public Health Madison Wisconsin USA; ^3^ Rush Alzheimer's Disease Center Rush University Medical Center Chicago Illinois USA; ^4^ Department of Neurological Sciences Rush University Medical Center Chicago Illinois USA; ^5^ Department of Neurology University of Wisconsin–Madison School of Medicine and Public Health Madison Wisconsin USA; ^6^ Department of Medical Physics University of Wisconsin–Madison School of Medicine and Public Health Madison Wisconsin USA; ^7^ Department of Neurology and Neurological Sciences Stanford University Palo Alto California USA; ^8^ Department of Radiology University of Wisconsin–Madison School of Medicine and Public Health Madison Wisconsin USA

**Keywords:** amyloid, Clinical Dementia Rating, preclinical Alzheimer's disease, white matter hyperintensities

## Abstract

**INTRODUCTION:**

Amyloid (A) and vascular (V) pathologies often co‐occur and progress over decades. We leveraged chronicity, defined as the years above a biomarker‐positivity threshold, to examine how the timing of A and V relates to cognitive decline.

**METHODS:**

We modeled Clinical Dementia Rating–Sum of Boxes (CDR‐SB) trajectories in *n* = 558 participants with [C‐11] Pittsburgh compound B positron emission tomography (PET), magnetic resonance imaging–derived white matter hyperintensities (WMHs), and longitudinal CDR assessments. In *n* = 500 with MK6240 PET, we tested whether tau mediates A–V associations with CDR‐SB in a moderated mediation framework.

**RESULTS:**

Whether biomarker “burden” was modeled as chronicity (A+years, V+years), or estimated amyloid and WMH at CDR visits, significant interactions showed a synergistic effect of WMHs and amyloid on accelerated CDR‐SB trajectories. Tau significantly mediated these associations.

**DISCUSSION:**

Operationalizing chronicity clarifies how long individuals have exceeded A and V thresholds and improves clinical interpretability. WMH accumulation exacerbates amyloid‐related cognitive decline. Longitudinal tau imaging could further inform staging and intervention timing.

**Highlights:**

- Longer amyloid (A) and vascular disease (V) chronicity were associated with faster cognitive decline, emphasizing the importance of considering chronic exposure to these pathologies.
- Individuals with higher V burden experienced a steeper decline in cognition in the presence of A pathology, highlighting the interaction between V and neurodegenerative processes.
- Progression from early to mild dementia was faster with greater white matter hyperintensity chronicity, even when A duration was held constant, supporting the idea that V pathology amplifies the clinical impact of amyloid in Alzheimer's disease.
- Tau accumulation played a significant mediating role in linking A and V burden to cognitive decline, suggesting that tau pathology is a critical downstream factor in symptom progression.
- Person‐level chronicity estimates of A and V provide a more precise understanding of cognitive decline trajectories, offering insights for early intervention strategies.

## 1 BACKGROUND

In the context of Alzheimer's disease (AD) and related dementias (ADRD), symptom progression to mild cognitive impairment (MCI) and dementia is often a confluence of underlying multiple coexisting pathologies, primarily AD pathology and vascular (V) disease.^1^, ^2^ Numerous studies demonstrate these two pathologies frequently co‐occur,^3^, ^4^, ^5^ leading to multi‐cause dementia, and complicating diagnosis and treatment.^6^

These pathologies often initiate at different ages.^7^, ^8^ For example, amyloid onset spans ≈ 40 years,^9^ and vascular pathology onset also shows substantial variability, with an estimated range of ≈ 30 years, as reported in our prior study of the V+ group.^10^ Understanding the timing of their onset could help better align individuals within specific disease biomarker stages and provide insight into years to dementia anchored to the onset age of each pathology. A study from the Mayo Clinic^11^ and our recent study^10^ indicated that although both amyloid deposition (A+) and white matter hyperintense lesion volume, a marker of cerebrovascular ischemic damage (V+), are each age related, their onset and trajectories are independent. Additionally, trajectories of V and A accumulation in these studies were highly predictable from the onset of biomarker positivity. Cognitive decline across multiple domains was most rapid when both V and A were concurrently present.

We use chronicity as a time‐based exposure: the estimated number of years an individual has spent above the biomarker‐positivity threshold. We apply this construct to both amyloid (amyloid chronicity) and white matter hyperintensities (WMHs) chronicity. Methodological details are provided in section 2.4. Building on this framework, our previous research^12^ demonstrated that longer A+ chronicity was associated with increased tau positron emission tomography (PET) accumulation and accelerated cognitive decline on a preclinical AD composite in cognitively unimpaired (CU) individuals at baseline. Tau pathology has also been shown to mediate the synergistic influence of vascular risk and amyloid beta on cognitive decline, reinforcing its role as a key factor in AD progression.^13^ Additionally, studies suggest that tau may moderate the relationship between amyloid and cognition, with higher tau burden amplifying the detrimental effects of amyloid pathology on cognition.^14^

Our prior work, using the Wisconsin Registry for Alzheimer's Prevention (WRAP) and other cohorts, showed that the sampled iterative local approximation (SILA) method effectively estimates person‐level amyloid^9^, ^14^ onset age and duration, and extended this framework to WMHs to estimate cerebrovascular onset timing.^10^ Other research groups have also demonstrated the ability to derive person‐level estimates of amyloid onset or A+ age using different mathematical methods,^12^, ^15^, ^16^, ^17^, ^18^, ^19^, ^20^ subsequently using these estimates to characterize clinical decline or biomarker development. For example, Schindler et al.^17^ showed that the onset of clinical symptoms, assessed by the Clinical Dementia Rating (CDR),^21^ was highly correlated with the age of amyloid onset. Birdsill et al.^22^ reported that amyloid chronicity (i.e., estimated years above the amyloid‐positivity threshold) was associated with CDR‐SB trajectories, with a dementia‐equivalent CDR rating occurring, on average, ≈ 24 years after amyloid onset; individuals who declined earlier than expected were older and had higher WMH volumes.^22^

Building on these prior findings, this study included individuals enrolled in either the WRAP or the Wisconsin Alzheimer's Disease Research Center (WADRC) and had two primary aims.

**RESEARCH IN CONTEXT**

1. **Systematic review**: The authors conducted a literature review using traditional sources (e.g., PubMed) to examine the temporal progression of Alzheimer's disease (AD) pathology, cerebrovascular disease pathology, and clinical symptom onset. The review focused on evidence related to the AD pathological cascade, including studies estimating amyloid onset. Recent findings on the relationships among AD biomarkers, ischemic vascular (V) changes, and cognition were also examined.
2. **Interpretation**: Our findings highlight individual variability in symptom onset relative to amyloid (A) and V positivity duration and provide initial evidence that tau mediates the relationship between A–V interactions and cognitive decline.
3. **Future directions**: This study provides a framework for understanding the complex interactions among AD and V etiologies and subsequent clinical progression. Future research should incorporate longitudinal tau imaging and expand to include other cerebrovascular indicators and biomarkers of other etiologies. Other cognitive outcomes may be sensitive to subtle decline. The findings should be validated in diverse cohorts using alternative cerebrovascular and AD biomarkers.

Aim 1: Examine trajectories of cognitive decline measured by harmonized CDR Sum of Boxes (CDR‐SB) scores in relation to the estimated years of WMH positivity (V+) and years of amyloid positivity (A+).

Aim 2: Investigate whether tau pathology mediates or moderates the association of WMHs and amyloid markers with CDR‐SB, thus clarifying the complex interplay among these key neuropathological factors in AD progression.

## 2 METHODS

### 2.1 Participants

Participants were drawn from the WRAP and WADRC. Both cohorts are longitudinal studies that are enriched for risk of dementia by virtue of oversampling for a parental family history of AD. WRAP recruitment began in 2001; all participants were dementia free at WRAP study baseline, and participants returned approximately biannually for follow‐up visits.^23^ The WADRC comprises three longitudinal cohorts at varying stages of disease progression (younger CU, older CU, MCI, and dementia), with recruitment starting in 2009. Participants return annually for follow‐up visits, except for CU individuals < 65, who are assessed biennially. A total of 2382 WRAP and WADRC participants had longitudinal neuropsychological assessments. Of these, 738 participants had at least one amyloid PET scan and one harmonized CDR‐SB assessment, and 1069 had at least one magnetic resonance imaging (MRI)‐derived WMH volume and one harmonized CDR‐SB assessment. The final analytic dataset for Aim 1 included 558 participants who had all three measures. This sample included 357 participants from the WRAP cohort and 201 from the WADRC cohort. To be included in Aim 2, participants were required to have also tau PET data (*n* = 500). A detailed flowchart is presented in Figure S1 in supporting information. All subjects provided informed consent, and study procedures were approved by the University of Wisconsin–Madison Institutional Review Board and conducted in accordance with the Declaration of Helsinki.

### 2.2 Neuropsychological assessment protocol

Participants in each cohort underwent overlapping neuropsychological batteries at each biennial visit. WRAP includes cognitive measurement at ≈ 2 year intervals and the comprehensive battery is described elsewhere.^23^ Both studies include study partner reports. In the WADRC, the CDR^21^ is used to obtain a CDR‐SB score. In WRAP, a validated, harmonized CDR‐SB is obtained as follows: all study partners are asked to complete a Quick Dementia Rating System (QDRS) screener.^24^ If the QDRS is elevated above a level corresponding to a CDR global score = 0, study partners are asked to complete a CDR; to maintain blindness to participant status, some participants with normal QDRS scores are also selected for CDR completion. If a CDR has been completed, the CDR‐SB is used in the CDR‐SB harmonized score; if only a QDRS is available, the sum of the first six items is used as the harmonized CDR‐SB score (for details, see Berman et al.^25^ and Huang et al.^26^). Among the 357 WRAP participants included in Aim 1, 315 (88%) had QDRS‐derived scores (sum of the first six items), and 42 (12%) had CDR‐SB scores at their most recent visit. For the remainder of the paper, CDR‐SB refers to the combined WADRC and WRAP harmonized CDR‐SB variables. The CDR‐SB ranges from 0 to 18 and is calculated by summing scores across six domains, with higher scores indicating greater impairment. Dementia severity was classified using CDR‐SB cutoffs defined in a previous study: 2.5–4.0 (very mild), 4.5–9.0 (mild), 9.5–15.5 (moderate), and 16.0–18.0 (severe).^27^

### 2.3 Neuroimaging

All participants included in these analyses underwent amyloid PET and MRI imaging. In Aim 2, a subset of participants who also had tau PET imaging was included in the analysis. Amyloid PET procedures are described in section 2.3.1, MRI procedures and WMH quantification are described in section 2.3.2, and tau PET procedures are described in section 2.3.3.

#### 2.3.1 PET amyloid

Amyloid burden was assessed with [C‐11] Pittsburgh compound B (PiB) PET imaging. Details for PET acquisition, processing, quantification, and analysis methods have been described elsewhere.^28^, ^29^ T1‐weighted MRI was used for tissue class and anatomical segmentation using SPM12. A multi‐region of interest (ROI) distribution volume ratio (DVR) value (DVR derived from a 0–70 minute dynamic scan) is described in Betthauser et al.^9^ For translatability, DVR values were also linearly translated to Centiloids (CLs) using the equation: equivalent CLs = 148.33×DVR‐154.96.^9^ In these analyses, PiB positivity (A+) was defined as a global 11C‐PiB DVR ≥ 1.16 (≈17 Equivalent CL^9^). Longitudinal PET data were included in the analysis if people had at least one WMH value.

#### 2.3.2 MRI assessment of WMHs

Participants underwent 3T MRI (GE Healthcare MR750 or Premier), including volumetric (3D) T2 fluid attenuated inversion recovery (FLAIR) and T1‐weighted acquisitions. T1‐weighted images were bias corrected, tissue class segmented, and spatially normalized to MNI152 standard space (SPM12; www.fil.ion.ucl.ac.uk/spm). WMH lesion volume in milliliters (mL) was segmented and summed using the automated lesion prediction algorithm (LPA) from the Lesion Segmentation Tool version 3.0.0 for SPM12 in MATLAB (MATLAB v.R2018b; MathWorks) from T2‐weighted FLAIR scans, and output as lesion probability maps. Detailed methods for MRI acquisition, processing, quantification, and analysis, and WMH volume acquisition, were previously reported.^10^, ^28^ Raw WMH volume was normalized to total intracranial volume (TICV; sum of gray matter [GM], white matter, and cerebrospinal fluid [CSF] from SPM segmentation), expressed as a percentage of TICV, and scaled to the cohort mean TICV to obtain an adjusted volume in mL (adjusted WMH = WMH/TICV x mean [TICV]). WMH positivity (V+) was defined as adjusted WMH ≥ 2.06 mL, a threshold derived from a two‐class Gaussian mixture model in a larger overlapping sample (*n* = 931), with the component‐density intersection as the cut‐point.^10^ Longitudinal WMH data were included in the analysis if participants had at least one amyloid PET.

#### 2.3.3 Tau PET

Tau burden was assessed with [^18^F]‐MK‐6240 (florquinitau) PET imaging. Details for radioligand synthesis, PET acquisition, processing, quantification, and analysis methods have been described previously.^29^ Tau burden was quantified as the standardized uptake value ratio (SUVR, 70–90 minutes post‐injection, inferior cerebellum GM reference region)^29^ in a temporal meta ROI (MTC) encompassing the entorhinal cortex, amygdala, parahippocampal gyrus, fusiform gyrus, inferior and middle temporal gyri regions. Tau positivity was ascertained using a previously defined threshold (temporal meta ROI MK6240 SUVR > 1.30; MK ±).^14^

### 2.4 PET amyloid burden and WMH burden

Biomarker burden was operationalized either as chronicity (duration above positivity) or as level (continuous value). To estimate the onset age of WMH and PET amyloid, these biomarkers were binarized into normal and abnormal categories using the cutoffs described in sections 2.3.1 and 2.3.2. We then applied the SILA method (https://github.com/Betthauser‐Neuro‐Lab/SILA‐AD‐Biomarker) to align biomarker levels to a duration scale, on which year 0 represents the threshold for biomarker positivity. SILA methodology and its validation for amyloid are detailed in Betthauser et al.;^9^ we have also applied and validated SILA for MRI‐derived WMH in an independent analysis from our group.^10^

Briefly, the SILA algorithm applies discrete sampling to model the relationships between PET amyloid levels and amyloid accumulation rates, as well as between WMH levels and WMH accumulation rates. Numerical smoothing using robust locally estimated scatterplot smoothing (LOESS) and the Euler method is used to integrate these data and generate non‐parametric PET amyloid and WMH versus duration curves. The zero time‐point represents the biomarker positivity threshold.

For each participant, an index scan anchors alignment to the cohort trajectory. Onset age is obtained by solving the biomarker level–duration curve at the observed level and subtracting the resulting duration from scan age. Chronicity at a CDR visit equals age at that visit minus onset age. Using all quality control (QC)‐passed scans, we computed at each CDR visit: chronicity(t) (years above positivity), and level(t) (PiB DVR or adjusted WMH mL). If no same‐day imaging was available, SILA provided antecedent/prospective estimates on the participant‐specific curve at the visit date. When multiple scans existed, earlier and later scans contributed to alignment and were used for prediction checks. However, because the SILA model is not designed to handle data with a floor effect, the lower bound of WMH estimates can exhibit a slight negative slope. This occurs due to a gradual slope at the lower end of the model, where the mean WMH rate remains slightly positive. To address this, any negative WMH estimates were truncated to zero, as they effectively hold the same interpretation.

### 2.5 Vascular measures

We report systolic and diastolic blood pressure (SBP and DBP; mmHg), body mass index (BMI; kg/m^2^), total cholesterol (mg/dL), and creatinine (mg/dL) because these variables have high availability. Prespecified QC thresholds set implausible values to missing (for example, SBP < 70 or > 300 mmHg; DBP < 30 or > 200 mmHg; BMI < 12 or > 70 kg/m^2^; total cholesterol < 70 or > 400 mg/dL; creatinine < 0.2 or > 6.0 mg/dL).

### 2.6 Statistical analyses

Analyses were conducted in R (v4.0.2; R Core Team, 2020), except for SILA modeling, which was performed in MATLAB 2024b. Descriptive statistics of all participants with CDR‐SB, PET amyloid, and WMH biomarkers are presented as mean (standard deviation [SD]) for normally distributed and median (25th, 75th percentile) for non‐normally distributed continuous data, and as *n* (%) for categorical variables.

#### 2.6.1 Aim 1: Examine trajectories of CDR‐SB, relative to various time and biomarker parameterizations

First, in the primary model set, we used mixed effects models (all models included a random intercept; random slopes were retained only when significant prior to adding fixed effects) to examine whether baseline WMH chronicity modifies the association between time (modeled as estimated amyloid chronicity at each CDR assessment) and CDR‐SB trajectories. Baseline age in years at CDR‐SB assessment was adjusted in the model, centered at age 65. Sex and education level (< bachelor of arts degree [BA] vs. ≥ BA) were included in the model as covariates. We first ran a base model (Model 1a) including random effects, time‐varying amyloid chronicity (retaining significant polynomial terms), baseline CDR‐SB age, and covariates. Next, we added baseline WMH chronicity x amyloid chronicity to the base model and retained it for subsequent models if it was significant (*p* < .05; Model 1b). Although chronicity is our primary time scale, non‐positive chronicity values in biomarker‐negative participants are less interpretable than biomarker levels. To provide a burden measure that is continuous across the full cohort and directly comparable to common clinical metrics, we prespecified parallel level‐based models. In Model 1c, we substituted SILA‐estimated PiB DVR at each CDR visit for amyloid chronicity and baseline SILA‐estimated WMH volume for baseline WMH chronicity (retaining the same covariates and random‐effects structure). These parallel models allow us to verify that the amyloid × WMH association with CDR‐SB holds when biomarker burden is expressed as current level rather than time above threshold, while the chronicity models preserve the time‐based clinical interpretation.

Similarly, we used a secondary model set to examine whether baseline amyloid chronicity modifies the association between estimated WMH chronicity at each CDR and CDR‐SB trajectories (i.e., this model set is parallel to the primary set but switches the time‐varying predictor to WMH chronicity). Model 2a (base model) included random effects, time as estimated WMH chronicity (retaining significant polynomial terms), baseline age in years at CDR‐SB assessment, sex, and education level (< BA vs. ≥ BA). Model 2b added the baseline amyloid chronicity x WMH chronicity interaction to the base model and then removed it if not significant. Model 2c also included the WMH/PiB interaction, replacing time‐varying WMH chronicity with estimated WMH at each CDR and baseline PiB chronicity with estimated PiB DVR at baseline CDR‐SB assessment.

Last, to compare these results to a model that characterizes patterns in a way that is more typical for clinicians, we ran a final linear mixed effects model for Aim 1 that used age at CDR assessments as the time operationalization (centered at age 65 and retaining significant polynomial terms as before) and a four‐group biomarker status variable based on CDR baseline amyloid and WMH status (PiB–/WMH– = reference group) and the interaction between the two (model 3); the same random effects and covariates were included here as in the prior models.

Model sets 1 and 2 compare two time‐varying parameterizations: current SILA‐estimated biomarker level at each visit (Models 1c, 2c) versus SILA‐estimated chronicity at each visit (Models 1b, 2b). Because these parameterizations target different estimands and are not strictly nested, we used Akaike information criterion‐corrected (AICc) to compare in‐sample fit. We compared the model fits of Models 1a–c, 2a–c using AICc; we report results for the best‐fitting model and any others with ∆AICcs < 2 for each biomarker, as these models represent a similarly adequate fit.^30^ AICc was calculated using the AICcmodavg R package.^31^ When significant interactions were identified, we used the emmeans R package^32^ to estimate simple slopes and 95% confidence intervals (CIs) at selected values of chronicity or biomarker levels (e.g., chronicities 0, 10, and 20). In Model 1b, slopes of amyloid chronicity at CDR were estimated across WMH chronicity strata defined by medians within ≤ 0, 0 to 10, and > 10 year groups. To enhance clinical interpretability, we converted the model‐predicted annual CDR‐SB change for selected PiB × WMH strata into a model‐implied years from 0.5 (within the normal range) to 6.5 (representing mid‐range mild dementia) as $\frac{6.5-0.5}{\Delta CDR-SB/year}$. Model diagnostics were examined and indicated that residuals were reasonably well distributed across the predicted range, supporting the validity of these longitudinal predictions. Using the fitted model, we generated predicted values for nine PiB × WMH chronicity subgroup combinations (< 0, 0–10, and > 10 years), holding all other covariates constant (female sex, college education, and median age). Representative median values were used for each chronicity group. In Model 1c, slopes of estimated PiB DVR were examined across WMH burden strata using WMH volume cut points at 2.06 and 10. Model 2b estimated slopes of WMH chronicity across PiB chronicity strata (≤ 0, 0–10, > 10), and Model 2c estimated slopes of WMH across PiB DVR strata (≤1.16, 1.16–1.5, > 1.5). All other covariates were held constant at their reference levels or sample medians.

To compare amyloid and WMH contributions on a common scale, we performed block‐wise tests within the best‐fitting specification (e.g., Model 1c). Using maximum likelihood, we fit the full model and then removed either the amyloid block (PiB level, its quadratic term, and all interactions with WMH) or the WMH block (WMH level and its interactions with PiB). We report likelihood‐ratio tests, ΔAICc within the same model, and incremental marginal $R^{2}$ (Nakagawa) for each removal. As a robustness check, we computed block‐wise permutation importance with *B* = 200 iterations: for the amyloid block, PiB was permuted within person across visits and the polynomial and interactions recomputed; for the WMH block, WMH (constant within person) was reassigned across persons at the ID level and propagated within person before recomputing interactions. For each block, we summarize the median and interquartile range (IQR) of Δ AICc, $\Delta R_{marg}^{2}$, and Δ logLik relative to the original fit.

#### 2.6.2 Aim 2: Investigate the mediation effect of tau relative to WMH and Amyloid associations with CDR‐SB

We extended the best‐fitting models (Model b and Model c) from both primary and secondary model sets in Aim 1 by incorporating the first available tau pathology as a potential mediator. Because the age at the last available CDR assessment was closest to the age at the first available tau measurement, the last CDR score was used as the outcome instead of longitudinal trajectories. In the primary mediation models, amyloid chronicity or estimated amyloid at the last CDR served as the predictor (models 4b and 4c), with baseline WMH chronicity or estimated baseline WMH included as the moderator. These models correspond to models 1b and 1c from the primary model set in Aim 1. In the secondary mediation models (models 5b and 5c), WMH chronicity or estimated WMH at the last CDR served as the predictor, and baseline amyloid chronicity or estimated baseline amyloid were included as moderators, reflecting models 2b and 2c from Aim 1. All models included sex, education level, and the time difference between the last CDR and baseline tau as covariates. To support clinical interpretation, we first estimated predicted tau values and 95% CIs for subgroups defined by PiB and WMH chronicity levels (< 0, 0–5, > 5 years), using the fitted model with median values for each subgroup and holding other covariates constant. Predicted values were summarized and visualized using a forest plot. Next, to evaluate the clinical impact of tau on cognitive outcomes, we used Model 4b to estimate predicted annual changes in CDR‐SB for the same nine PiB × WMH subgroup combinations. Predictions were generated under two conditions: (1) using median tau SUVR values for each subgroup, and (2) setting tau SUVR to zero to approximate a scenario without tau burden. The difference in predicted CDR‐SB change between these two scenarios was interpreted as the contribution of tau to clinical worsening. CIs were derived from model‐based standard errors and visualized using grouped bar plots.

In an exploratory analysis, we assessed whether tau pathology mediated the relationship between amyloid, WMH, and cognitive change using annualized CDR‐SB change as the outcome. In models 6b and 6c, amyloid chronicity or estimated amyloid at baseline tau served as predictors, and WMH chronicity or estimated WMH at baseline tau were included as moderators. In models 7b and 7c, the roles were reversed, with WMH measures as predictors and amyloid measures as moderators.

All mediation analyses were conducted using a moderated mediation framework implemented via the mediation package in R.^33^ The significance of indirect effects was evaluated using non‐parametric bootstrapping to characterize the mediating role of tau pathology in the associations among amyloid, WMH, and cognitive outcomes.

## 3 RESULTS

The mean (SD) ages at baseline and last CDR assessments were 62.63 (7.24) and 67.26 (8.14) years, respectively. Amyloid and WMH chronicity at CDR visits (or the SILA‐estimated levels) were derived from *n* = 738 PET scans and *n* = 1069 MRI scans. The PET series spanned a median of 6.6 years (IQR 4.4–8.5), with 666/738 (90.2%) participants having ≥ 2 scans. The MRI series spanned a median of 6.3 years (IQR 4.2–8.5), with 992/1069 (92.8%) having ≥ 2 scans. At baseline CDR, there were 413 PiB−/WMH−, 40 PiB−/WMH+, 85 PiB+/WMH−, and 20 PiB+/WMH+ participants, based on estimated PiB amyloid and WMH burden at baseline using SILA methods. Participant characteristics overall and by group are summarized in Table 1. Briefly, the sample included more women than men, and the average age at first WMH and PiB scans was in the late 60s. Most people had a CDR Global score = 0 at baseline (i.e., indicating unimpaired), and most had three or more CDR measurements (42 [7.5%] had only one CDR measurement). Coverage of vascular risk factors was high for the variables reported in Table 1: SBP/DBP 550/558 (98.6%), BMI 555/558 (99.5%), total cholesterol 551/558 (98.7%), and creatinine 525/558 (94.1%). Additional vascular variables were not reported due to non‐harmonized ascertainment and substantial missingness. At baseline CDR, 373/558 (66.8%) had any estimated adjusted WMH; within‐cohort distributions of adjusted WMH are shown in Table 1. The sample exhibited a maximum of 22 years of estimated amyloid chronicity and 16 years of estimated WMH chronicity at first CDR. The median age of A+ onset in the A+ group was 63 years (range: 40–80), and the median age of V+ onset in the V+ group was 68 years (range: 51–85).

**TABLE 1:** Analytic sample characteristics.

Characteristic	Overall (*N* = 558)	PiB‐&WMH– (*N* = 413)	PiB‐&WMH+ (*N* = 40)	PiB+&WMH– (*N* = 85)	PiB+&WMH+ (*N* = 20)
Age at first CDR, years	62.63 (7.24)	60.91 (6.74)	69.18 (6.13)	66.24 (6.49)	69.73 (4.50)
Age at first PiB, years	65.42 (7.78)	63.93 (7.44)	71.36 (7.21)	68.40 (7.31)	71.44 (5.56)
Age at first WMH, years	65.42 (7.78)	63.93 (7.44)	71.36 (7.21)	68.40 (7.31)	71.44 (5.56)
Age at first tau,^a^ years	67.45 (7.26)	66.07 (7.12)	71.74 (6.16)	70.82 (6.47)	72.81 (4.44)
Female, *n* (%)	380 (68.1)	281 (68.0)	30 (75.0)	54 (63.5)	15 (75.0)
Non‐Hispanic White, *n* (%)	496 (88.9)	370 (88.9)	34 (85.0)	75 (88.2)	17 (85.0)
College, *n* (%)	381 (68.3)	289 (70.0)	23 (57.5)	55 (64.7)	14 (70.0)
WRAP cohort, *n* (%)	357 (64.0)	265 (64.2)	24 (60.0)	60 (70.6)	8 (40.0)
*APOE* ε4 carriers,^b^ *n* (%)	208 (39.7)	129 (33.4)	5 (13.5)	58 (70.7)	16 (84.2)
CDR‐SB at baseline, points (median [IQR], range)	0.00 [0.00, 0.00], (0–4.5)	0.00 [0.00, 0.00], (0–4.5)	0.00 [0.00, 0.50], (0–2)	0.00 [0.00, 0.50], (0–3.5)	0.50 [0.00, 1.75], (0–4)
Years of CDR follow‐up, years (median [IQR])	6.93 [4.94, 9.00]	7.11 [5.07, 9.07]	5.70 [3.39, 7.23]	6.93 [4.99, 8.91]	5.69 [4.81, 7.07]
Number of CDR assessments, *n* (median [IQR])	4.00 [3.00, 5.00]	4.00 [3.00, 5.00]	3.50 [2.00, 6.25]	4.00 [3.00, 5.00]	5.00 [2.00, 7.00]
Estimated age of PiB positivity, years (median [IQR])	72.68 [65.51, 84.24]	75.44 [68.65, 86.69]	82.47 [77.44, 101.13]	57.69 [52.39, 64.54]	60.63 [54.47, 65.19]
Estimated age of WMH positivity, years (median [IQR])	72.03 [67.95, 77.96]	72.25 [68.36, 77.90]	64.99 [62.05, 70.59]	75.76 [71.39, 81.64]	64.66 [61.88, 66.15]
Estimated PET amyloid at first CDR, DVR (median [IQR])	1.06 [1.03, 1.10]	1.05 [1.03, 1.07]	1.05 [1.02, 1.08]	1.38 [1.27, 1.59]	1.47 [1.25, 1.68]
Estimated WMH at first CDR, mL (adjusted) (median [IQR])	0.27 [0.00, 0.63]	0.15 [0.00, 0.47]	3.27 [2.77, 4.39]	0.34 [0.00, 0.61]	4.24 [3.32, 9.89]
Amyloid positive at last PiB, *n* (%)	182 (32.6)	74 (17.9)	3 (7.5)	85 (100.0)	20 (100.0)
Amyloid chronicity at first CDR, years	2.56 (7.83)	−4.88 (2.53)	−4.32 (3.34)	7.77 (5.36)	8.99 (6.54)
WMH positive at last WMH, *n* (%)	124 (22.2)	49 (11.9)	40 (100.0)	15 (17.6)	20 (100.0)
WMH chronicity at first CDR, years	0.21 (4.67)	−3.48 (2.37)	3.16 (2.42)	−2.76 (2.47)	5.58 (4.46)
MTC at baseline,^a^ SUVR (median [IQR])	1.11 [1.03, 1.19]	1.10 [1.03, 1.16]	1.10 [1.01, 1.13]	1.26 [1.09, 1.64]	1.31 [1.11, 2.64]
Baseline CDR global, *n* (%)					
0	475 (85.1)	369 (89.3)	30 (75.0)	65 (76.5)	11 (55.0)
0.5	82 (14.7)	43 (10.4)	10 (25.0)	20 (23.5)	9 (45.0)
1	1 (0.2)	1 (0.2)	0 (0.0)	0 (0.0)	0 (0.0)
SBP,^c^ mmHg	125.06 (16.14)	123.83 (16.11)	134.26 (15.56)	125.29 (15.38)	131.63 (14.66)
DBP,^c^ mmHg	74.47 (9.58)	74.12 (9.76)	77.82 (7.94)	73.78 (9.74)	78.11 (5.73)
BMI,^d^ kg/m^2^	27.82 (5.74)	27.92 (5.93)	29.20 (5.46)	26.83 (4.30)	27.05 (6.98)
Total cholesterol,^e^ mg/dL	202.01 (36.74)	200.89 (35.98)	218.18 (45.52)	201.87 (31.46)	193.25 (46.47)
Creatinine,^f^ mg/dL	0.90 (0.18)	0.89 (0.17)	0.91 (0.16)	0.93 (0.22)	0.92 (0.21)

Notes: Values are mean (SD) unless otherwise stated; medians are shown as median [IQR]; counts as *n* (%). Units: years for all age and chronicity variables; points for CDR‐SB; DVR (unitless) for PET amyloid; SUVR (unitless) for tau (MTC); mL for WMH volume. Estimated WMH is adjusted to total intracranial volume (TICV) using adjusted WMH = (raw WMH / TICV) × mean (TICV). Positivity thresholds: PiB+ = DVR ≥ 1.16; WMH+ (V+) = adjusted WMH ≥ 2.06 mL (data‐driven cut‐point from a two‐class Gaussian mixture in a larger overlapping sample; see section 2.3). Chronicity is expressed as years above the biomarker positivity threshold at the indicated visit. Column headers denote biomarker status at baseline CDR (PiB−/WMH−, PiB−/WMH+, PiB+/WMH−, PiB+/WMH+).

Abbreviations: *APOE*, apolipoprotein E; BMI, body mass index; CDR, Clinical Dementia Rating; CDR‐SB, Clinical Dementia Rating Sum of Boxes; DBP, diastolic blood pressure; DVR, distribution volume ratio; IQR, interquartile range; MTC, meta‐temporal composites; PET, positron emission tomography; PiB, Pittsburgh compound B; SBP, systolic blood pressure; SD, standard deviation; SUVR, standardized uptake value ratio; WMH, white matter hyperintensities; WRAP, Wisconsin Registry for Alzheimer's Prevention.

^a^ Sample size is *N* = 500.

^b^ Sample size is *N* = 524.

^c^ Sample size is *N* = 547.

^d^ Sample size is *N* = 555.

^e^ Sample size is *N* = 551.

^f^ Sample size is *N* = 525.

### 3.1 Aim 1 trajectories of CDR‐SB and relative to estimated years of V+ and A+

**FIGURE 1:**
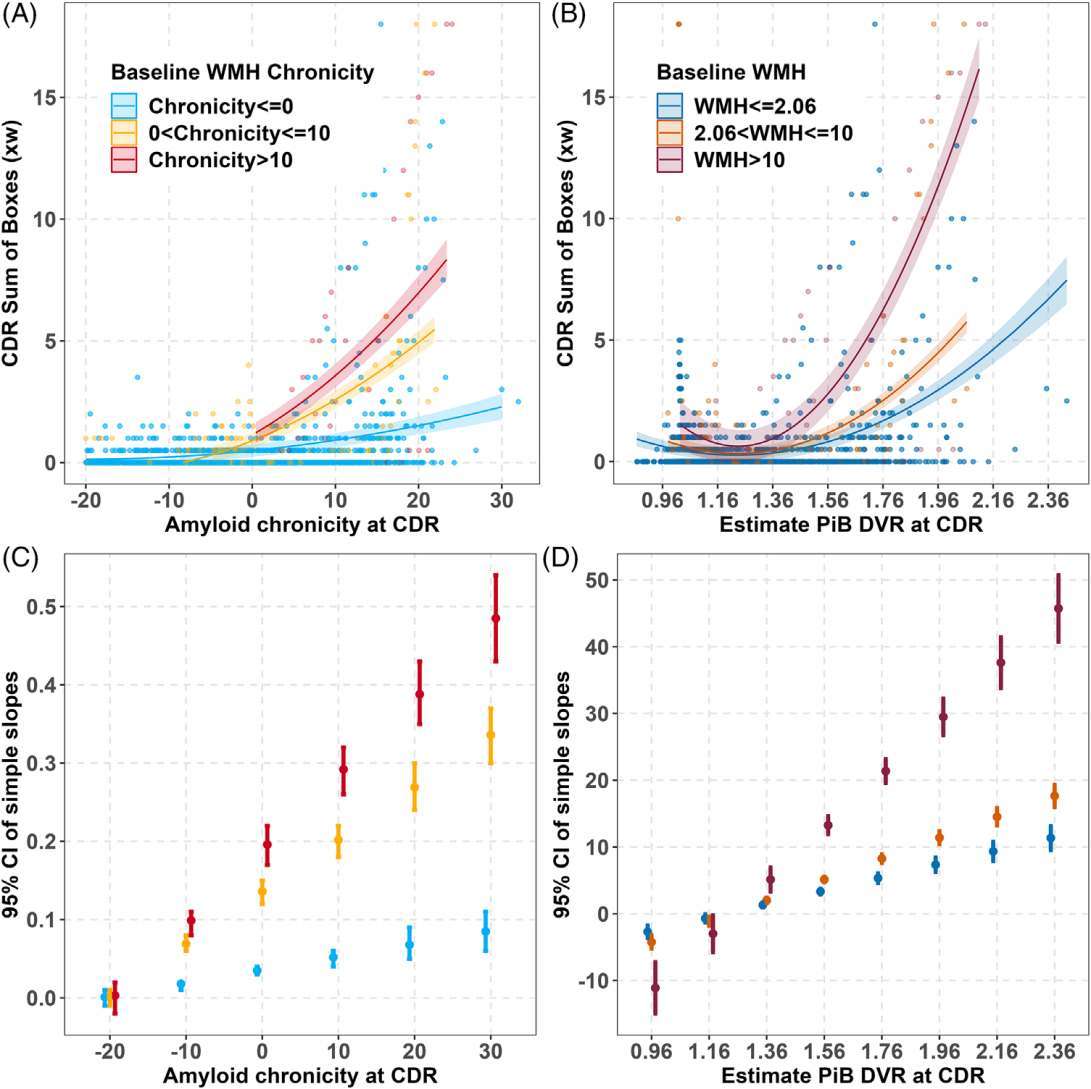
Interaction plot from mixed effect model of CDR Sum of Boxes and simple slopes (Model 1b and Model 1c from primary model set). A, Observed CDR Sum of Boxes (dots) for baseline WMH chronicity groups indicates that the higher WMH chronicity group increases fastest on average. This is an extension of Birdsill et al.^22^ (Model was CDR Sum of Boxes ∼ Baseline CDR age centered at 65 + amyloid chronicity at each CDR assessment x WMH chronicity at CDR baseline + quadratic amyloid chronicity + sex + education + random person – level intercepts.) Clinical Dementia Rating‐Sum of Boxes (CDR‐SB) was the Clinical Dementia Rating Scale (CDR; Morris^21^) itself whenever available or an analogous CDR‐SB derived from the Quick Dementia Rating Scale (QDRS, Galvin;^24^ Berman et al.^25^). DVR derived from a 0 to 70 minute dynamic 11C‐PiB PET scan. Chronicity was calculated as age at CDR – onset age estimated from SILA. Baseline WMH chronicity depicted are: < 0, 0 to 10, and > 10. Patterns suggest WMH moderated the association between amyloid chronicity and CDR. B, Observed CDR Sum of Boxes (dots) for baseline WMH groups indicates that the higher WMH group increases fastest on average. (Model was CDR Sum of Boxes ∼ baseline CDR age centered at 60 + estimated PiB DVR at each CDR assessment using SILA centered at 1.16 x baseline WMH groups + quadratic estimated PiB DVR + sex + education + random person – level intercepts.) Baseline chronicity depicted are: < 2.06, 2.06 to 10, and > 10. Side note: The Spearman correlation between estimated PiB DVR and amyloid chronicity at CDR is 0.999 when considering the full range of DVRs and chronicity estimates and 0.999 when considering only those with estimated PiB DVR ge 1.16 (in the A+ range). C, Simple slopes for baseline WMH chronicity groups of Model 2 from the primary model set. D, Simple slopes for baseline WMH groups of Model 3 from the primary model set. CDR, Clinical Dementia Rating; CI, confidence interval; DVR, distribution volume ratio; PET, positron emission tomography; PiB, Pittsburgh compound B; SILA, sampled iterative local approximation; WMH, white matter hyperintensity.

**FIGURE 2:**
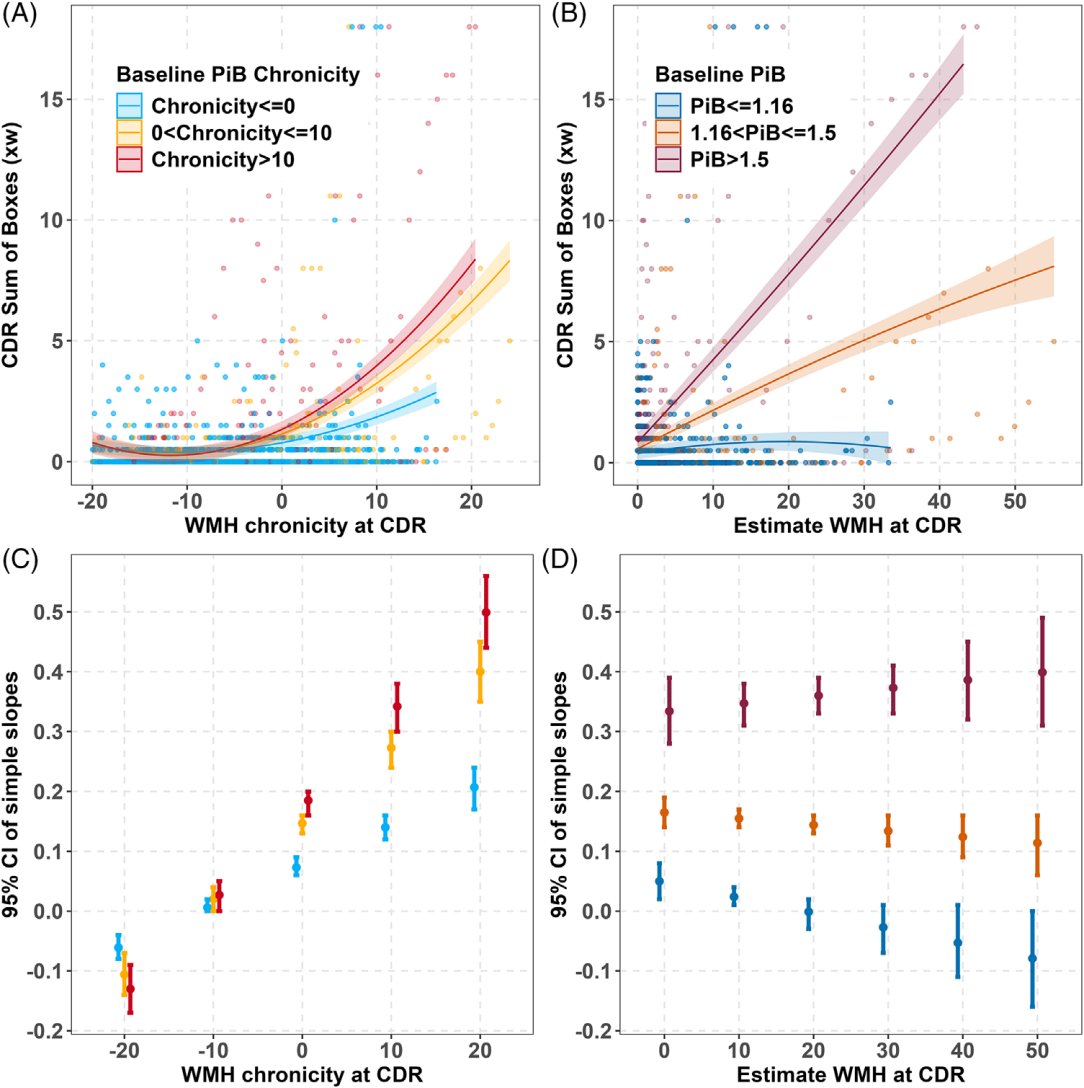
Interaction plot from mixed effect model of CDR Sum of Boxes and simple slopes (Model 2b and Model 2c from secondary model set). A, Observed CDR Sum of Boxes (dots) for baseline PiB chronicity groups indicate that the higher PiB chronicity group increases fastest on average. (Model was CDR Sum of Boxes ∼ Baseline CDR age centered at 65 + WMH chronicity at each CDR assessment x amyloid chronicity at CDR baseline + quadratic WMH chronicity + sex + education + random person‐level intercepts.) Chronicity was calculated as age at CDR – onset age estimated from SILA. Baseline PiB chronicity depicted are: < 0, 0 to 10, and > 10. Patterns suggest PiB moderated the association between WMH chronicity and CDR. B, Observed CDR Sum of Boxes (dots) for Baseline PiB groups indicates that the higher PiB group increases fastest on average. (Model was CDR Sum of Boxes ∼ Baseline CDR age centered at 60 + estimate WMH at each CDR assessment using SILA centered at 2.06 x baseline PiB DVR groups + quadratic estimate WMH + sex + education + random person‐level intercepts.) Baseline chronicity depicted are: < 1.16, 1.16 to 1.5, and > 1.5. C, Simple slopes for baseline WMH chronicity groups of Model 2 from the primary model set. D, Simple slopes for baseline WMH groups of Model 3 from the primary model set. CDR, Clinical Dementia Rating; CI, confidence interval; PiB, Pittsburgh compound B; SILA, sampled iterative local approximation; WMH, white matter hyperintensity.

Mixed‐effects analyses showed that both WMH burden and amyloid burden interact such that longer duration of each, or equivalently, higher levels of each, is associated with faster worsening in CDR‐SB. Figure 1 illustrates the baseline WMH x quadratic amyloid burden interactions for models 1b and 1c. Specifically, the estimated CDR‐SB trajectories related to PiB chronicity (Figure 1A) or estimated PiB DVR (Figure 1B) are shown for three baseline WMH levels representing WMH negative, 0 to 10 years of WMH+/moderate volumes, or > 10 years of WMH+/higher volumes, while simple slope estimates and CIs from the baseline WMH x PiB burden interactions are depicted in Figure 1C for baseline WMH chronicity and Figure 1D for estimated baseline WMH volume at specific amyloid chronicities/levels. Based on model‐predicted annual CDR‐SB change from Model 1b, individuals with 0 to 10 years of PiB chronicity and WMH chronicity < 0 years were model‐implied to take ≈ 20 years to move from a CDR‐SB of 0.5 to 6.5. This window shortened with increasing WMH chronicity: ≈ 5 years for WMH chronicity 0 to 10 years, and 3.5 years for WMH chronicity > 10 years. These quantities are translations of predicted slopes, not time‐to‐event estimates. These findings illustrate how coexisting vascular pathology may accelerate amyloid‐associated clinical progression and support the hypothesis that WMH burden modifies the clinical impact of AD pathology. Spaghetti plots of the CDR‐SB scores are shown in Figure S2 in supporting information versus the various time‐varying options and biomarker parameterizations that correspond to model sets 1, 2, and 3; corresponding model outputs are shown in Tables 2, 3, and S1 in supporting information. Figure 2 illustrates the parallel results for model set 2. Figure 2C and 2D show the corresponding simple slope estimates for Models 2b and 2c. Model 1c, which included estimated amyloid PiB DVR x baseline WMH, provided the best fit based on AICc comparisons (AICc = 7312.3), with a lower AICc compared to Model 1b (AICc = 7694.1) and Model 1a (AICc = 7902.8), indicating a better model fit (Table 2).

**TABLE 2:** Mixed effect model primary model set output.

	Model 1a			Model 1b			Model 1c		
Predictors	Estimates	CI	*p*	Estimates	CI	*p*	Estimates	CI	*p*
(Intercept)	0.69	0.43–0.96	**<0.001**	0.82	0.52–1.11	**<0.001**	0.35	0.11–0.58	**0.004**
Baseline CDR age	0.01	−0.01–0.02	0.282	−0.00	−0.02–0.01	0.649	0.00	−0.01–0.02	0.794
Female	−0.18	−0.40–0.05	0.120	−0.20	−0.41–0.01	0.063	−0.17	−0.37–0.03	0.101
College	−0.21	−0.43–0.01	0.066	−0.16	−0.37–0.05	0.139	−0.25	−0.45 to −0.04	**0.019**
PiB chronicity	0.06	0.05–0.07	**<0.001**	0.12	0.11–0.13	**<0.001**			
PiB chronicity^2	0.001	0.001–0.002	**<0.001**	0.00	0.00–0.00	**<0.001**			
Baseline WMH chronicity				0.03	0.01–0.04	**0.001**			
PiB chronicity × baseline WMH chronicity				0.007	0.006–0.008	**<0.001**			
Baseline WMH chronicity × PiB chronicity^2				0.0002	0.0001–0.0002	**<0.001**			
Estimate PiB							−0.95	−1.55 to −0.35	**0.002**
Estimate baseline WMH							0.03	−0.02–0.07	0.222
Estimate PiB^2							6.72	5.84–7.59	**<0.001**
Estimate PiB × baseline WMH							−0.14	−0.31–0.03	0.114
Estimate baseline WMH × PiB^2							0.91	0.71–1.10	**<0.001**
**Random effects**									
σ^2^	0.82			0.77			0.64		
τ_00_	1.30 _reggieid_			1.14 _reggieid_			1.10 _reggieid_		
ICC	0.61			0.60			0.63		
N	558 _reggieid_			558 _reggieid_			558 _reggieid_		
Observations	2567			2567			2567		
Marginal R^2^ / conditional R^2^	0.155 / 0.673			0.317 / 0.725			0.400 / 0.778		
AICc	7902.8			7694.1			7312.3		

Abbreviations: AICc, Akaike information criterion‐corrected; CDR, Clinical Dementia Rating; CDR‐SB, Clinical Dementia Rating‐Sum of Boxes; DVR, distribution volume ratio; ICC, intraclass correlation; IQR, interquartile range; MTC, meta‐temporal composites; PiB, Pittsburgh compound B; SUVR, standardized uptake value ratio; WMH, white matter hyperintensities.

*Model 1a: CDR SB = baseline CDR age + amyloid chronicity (and quadratic if sig) + covariates (sex, education) + random effects.

Model 1b: CDR SB = baseline CDR age + amyloid chronicity (and quadratic if sig) x baseline WMH chronicity + covariates (sex, education) + random effects.

Model 1c: CDR SB = baseline CDR age + estimated PiB DVR (and quadratic if sig) x baseline WMH + covariates (sex, education) + random effects.

Baseline CDR age is centered at age 65. Estimated PiB DVR is centered at DVR = 1.16, and estimated WMH is centered at adjusted WMH = 2.06.

**TABLE 3:** Mixed effect model secondary model set output.

	Model 2a			Model 2b			Model 2c		
Predictors	Estimates	CI	*p*	Estimates	CI	*p*	Estimates	CI	*p*
(Intercept)	0.81	0.55–1.07	**<0.001**	1.07	0.79–1.35	**<0.001**	0.66	0.43–0.90	**<0.001**
Baseline CDR age	0.00	−0.01–0.02	0.615	−0.00	−0.02–0.01	0.891	−0.01	−0.02–0.01	0.472
Female	−0.25	−0.48–−0.03	**0.029**	−0.29	−0.52– −0.07	**0.010**	−0.21	−0.42 to −0.00	**0.047**
College	−0.17	−0.40 – 0.05	0.134	−0.21	−0.44–0.01	0.060	−0.22	−0.43 to −0.01	**0.043**
WMH chronicity	0.09	0.08–0.10	**<0.001**	0.13	0.12–0.14	**<0.001**			
WMH chronicity^2	0.004	0.003–0.005	**<0.001**	0.01	0.00–0.01	**<0.001**			
Baseline PiB chronicity				0.02	0.01–0.03	**<0.001**			
Baseline PiB chronicity × WMH chronicity				0.004	0.003–0.005	**<0.001**			
Baseline PiB chronicity × WMH chronicity^2				0.0002	0.0001–0.0002	**<0.001**			
WMH							0.10	0.08–0.12	**<0.001**
WMH^2							−0.001	−0.002 to −0.000	**0.029**
Baseline PiB							1.73	1.17–2.29	**<0.001**
WMH × baseline PiB							0.48	0.38–0.58	**<0.001**
WMH^2 × baseline PiB							0.003	0.000–0.006	**0.048**
**Random effects**									
σ^2^	0.81			0.76			0.66		
τ_00_	1.33 _reggieid_			1.30 _reggieid_			1.18 _reggieid_		
ICC	0.62			0.63			0.64		
N	558 _reggieid_			558 _reggieid_			558 _reggieid_		
Observations	2567			2567			2567		
Marginal R^2^ / conditional R^2^	0.145 / 0.677			0.268 / 0.731			0.442 / 0.800		
AICc	7883.5			7732.6			7410.8		

Abbreviations: AICc, Akaike information criterion‐corrected; CDR, Clinical Dementia Rating; CDR‐SB, Clinical Dementia Rating‐Sum of Boxes; DVR, distribution volume ratio; ICC, intraclass correlation; IQR, interquartile range; MTC, meta‐temporal composites; PiB, Pittsburgh compound B; SUVR, standardized uptake value ratio; WMH, white matter hyperintensities.

*Model 2a: CDR SB = baseline CDR age + WMH chronicity (and quadratic if sig)+ covariates (sex, education) + random effects.

Model 2b: CDR SB = baseline CDR age + WMH chronicity (and quadratic if sig) x baseline amyloid chronicity + covariates (sex, education) + random effects.

Model 2c: CDR SB = baseline CDR age + estimated WMH (and quadratic if sig) x baseline PiB DVR + covariates (sex, education) + random effects.

Baseline CDR age is centered at age 65. Estimated PiB DVR is centered at DVR = 1.16, and estimated WMH is centered at adjusted WMH = 2.06.

In the secondary model set, baseline amyloid chronicity similarly modified the effect of WMH chronicity at each CDR on cognitive decline, with Model 2c again demonstrating the best model fit in this set (AICc = 7410.8; Table 3). Using age as the time scale (Model 3), individuals with elevated amyloid and/or WMH at baseline CDR showed significantly faster worsening in CDR‐SB compared to the PiB–/WMH– group, with the greatest worsening observed in the PiB+/WMH+ group. Polynomial age terms were not significant and were excluded. Results were consistent with the burden‐based models and are detailed in Table S1.

Across model sets (Tables 2 and 3), baseline CDR age (centered at 65) was not significant after accounting for biomarker terms and random intercepts. The parallel level‐based models (1c, 2c) reproduced the same qualitative amyloid × WMH pattern seen in the chronicity models, indicating that the association between these biomarkers and CDR‐SB trajectories is robust to whether burden is parameterized as current level or time above positivity.

Model 1c had the lowest AICc. Beyond AICc, within‐model block tests showed larger losses when removing amyloid terms than when removing WMH terms. In Model 1c, dropping the amyloid block versus the WMH block yielded LRT: $\Delta \;x^{2}=738.65\;\mathrm{versus}\;276.28$, both P<.001; Δ AICc: 730.59 versus 270.23; $\Delta R_{marg}^{2}:0.300\;\mathrm{versus}\;0.151$ (Table S2 in supporting information). Permutation importance agreed: median Δ AICc and $\Delta R_{marg}^{2}$ were larger when permuting the amyloid block than the WMH block (Table S3 in supporting information). Together, these results indicate that, within these specifications, amyloid terms explain more variance in CDR‐SB change, while WMH remains a significant modifier of amyloid‐related decline.

### 3.2 Aim 2 mediation effect of tau relative to WMH and amyloid associations with CDR‐SB

**FIGURE 3:**
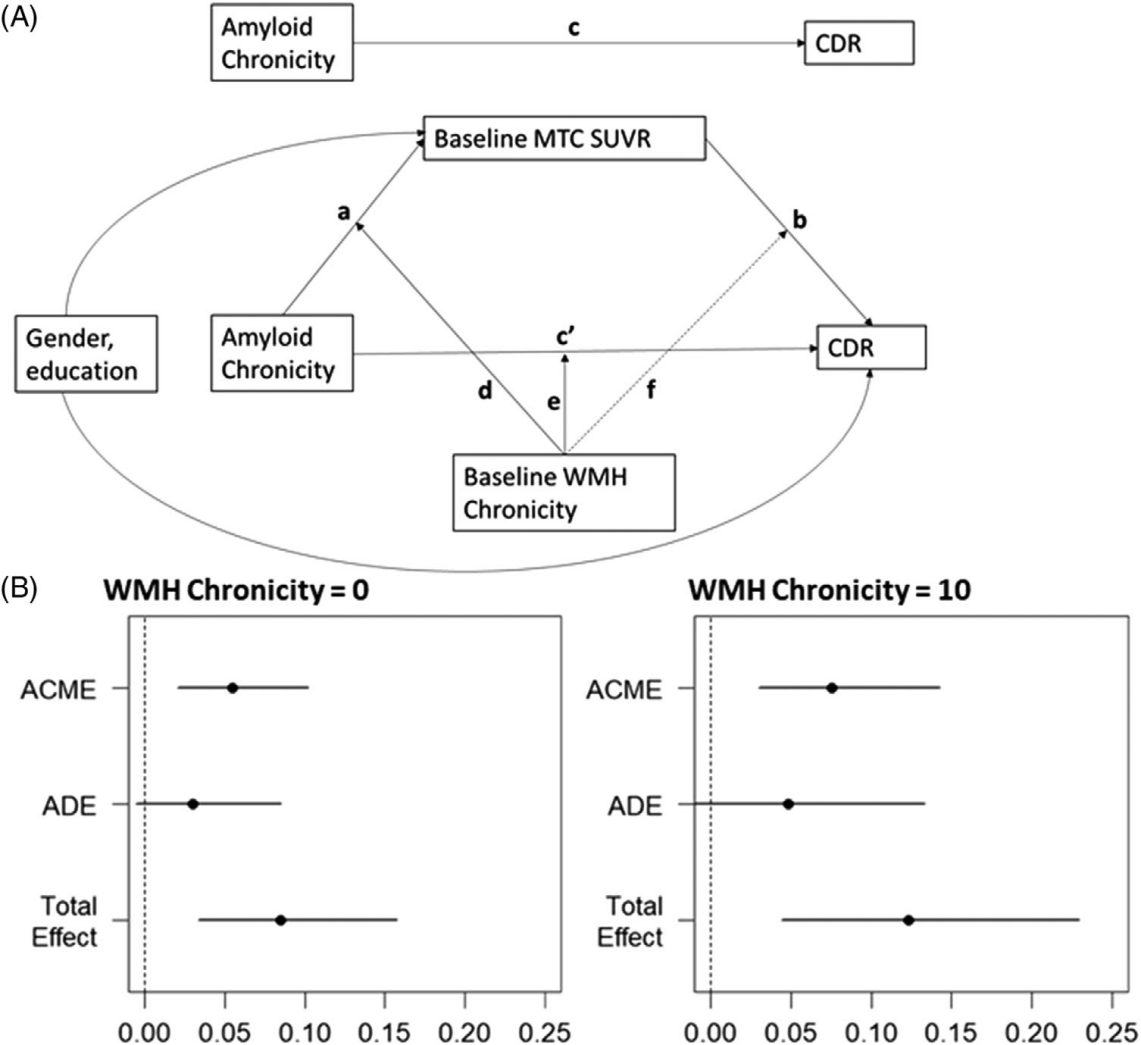
Mediated moderation analysis of last observed CDR‐SB (Model 4b). Meta‐temporal tau levels mediate the synergistic effect of WMH chronicity and amyloid chronicity on the last CDR‐SB. A, We modeled amyloid chronicity as a predictor, baseline WMH chronicity as a moderator, first available MTC tau as a mediator, and last CDR as an outcome, adjusting for baseline age, sex, education, and time between study baseline and first tau PET. Results indicate that MTC tau significantly mediated the effect of amyloid chronicity on CDR‐SB in individuals with low (B, left panel; *β* = 0.055 [0.021 to 0.10], *p* = 0.002) and high WMH chronicity (B, right panel; *β* = 0.075 [0.03 to 0.14], *p* < 0.001), accounting for 61% to 64% of the total effect. No statistically significant direct effect was observed. Tau was measured with florquinitau PET(MK6240; Meta‐temporal [MTC] SUVR composites derived from 70–90 minutes post‐injection). ACME, average causal mediation effects; ADE, average direct effect; CDR, Clinical Dementia Rating; CDR‐SB, Clinical Dementia Rating Sum of Boxes; CI, confidence interval; MTC, meta‐temporal composite; PiB, Pittsburgh compound B; PET, positron emission tomography; SUVR, standardized uptake volume ratio; WMH, white matter hyperintensity.

**FIGURE 4 alz70831-fig-0004:**
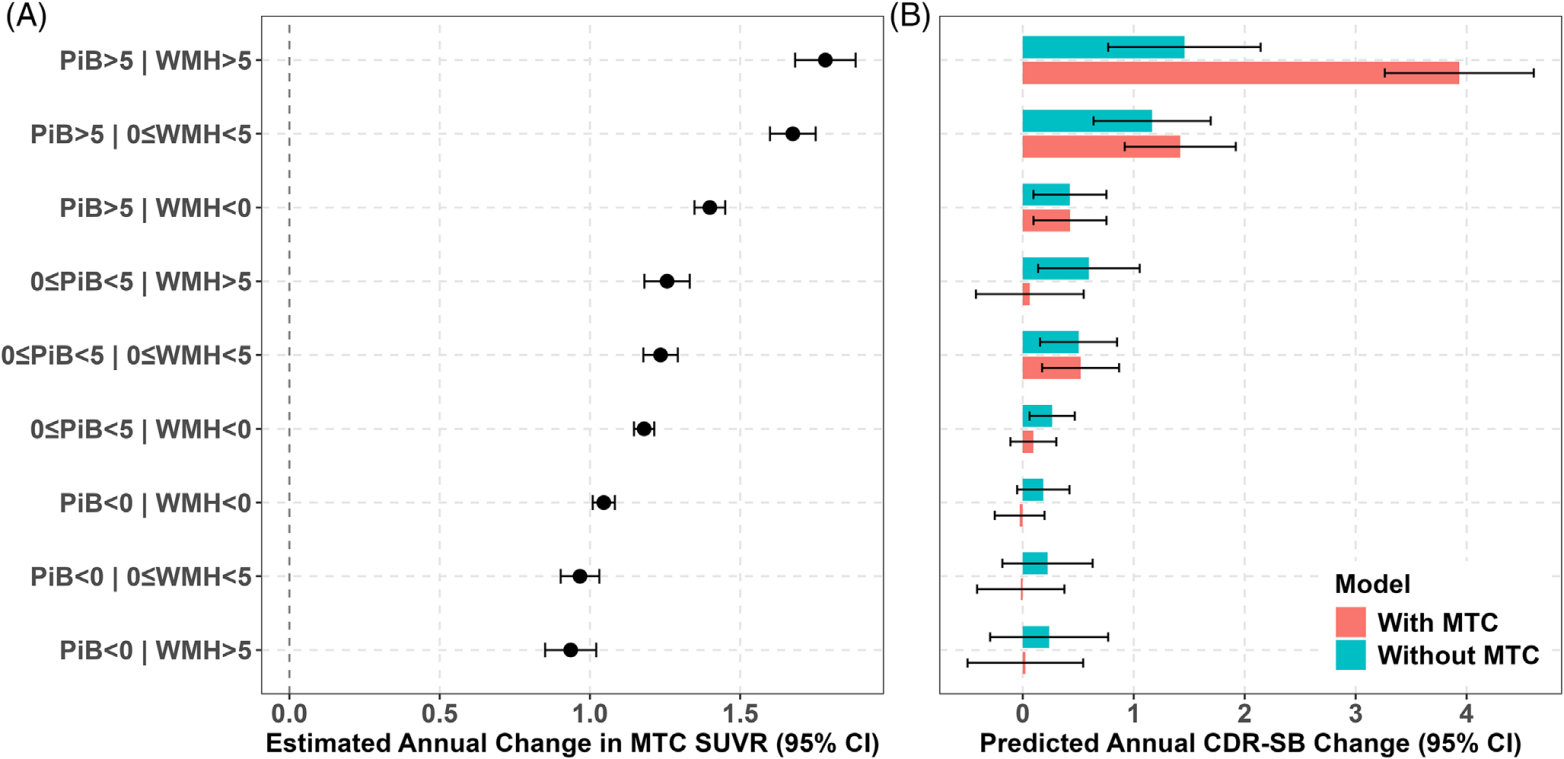
Estimated tau burden and its contribution to cognitive change across PiB and WMH chronicity subgroups (Model 4b). A, Estimated annual change in medial temporal cortex (MTC) SUVR by PiB and WMH chronicity subgroups (< 0, 0–5, > 5 years). Predictions were derived from a linear model using median PiB and WMH chronicity values for each subgroup, with all other covariates held constant (female sex, college education, and age difference between tau PET and last CDR = 2.035 years). Bars represent model‐based means and 95% confidence intervals. B, Predicted annual change in CDR‐SB for the same nine PiB × WMH chronicity subgroups, generated using Model 4b. Two sets of predictions are shown: (1) using subgroup‐specific median tau SUVR values (representing the “with tau” condition), and (2) setting tau SUVR to zero (representing the “without tau” condition). The tau‐related increment in CDR‐SB worsening is small at low amyloid or low WMH but increases when both exposures are > 5 years, consistent with tau mediating the combined effects of amyloid and WMH. Confidence intervals were estimated using model‐based standard errors. CDR, Clinical Dementia Rating; CDR‐SB, Clinical Dementia Rating Sum of Boxes; CI, confidence interval; MTC, meta‐temporal composite; PiB, Pittsburgh compound B; PET, positron emission tomography; SUVR, standardized uptake volume ratio; WMH, white matter hyperintensity.

Tau pathology, as measured by MK6240, a range of 0.80 to 3.75 for meta‐temporal composite (MTC) values, varied significantly across individuals, with greater tau burden observed in those with longer amyloid chronicity and higher WMH burden. The moderated mediation analysis revealed that MTC tau accumulation significantly mediated the synergistic effect of WMH and amyloid on the final CDR‐SB score. Figure 3A illustrates the mediation effects of tau pathology for Model 4b, where amyloid chronicity served as the predictor, baseline WMH chronicity as the moderator, first available MTC tau as the mediator, and last CDR as the outcome. This visualization highlights tau's role in the relationship between WMH, amyloid chronicity, and cognitive decline. Results indicate significant indirect effects of amyloid chronicity on CDR through MTC tau in individuals with low (Figure 3B, left panel; *β* = 0.055 [0.021 to 0.10], *p* = 0.002) and high WMH chronicity (Figure 3B, right panel; *β* = 0.075 [0.03 to 0.14], *p* < 0.001), accounting for 61% to 64% of the total effects. The direct effect was not statistically significant, suggesting that the association between amyloid chronicity and CDR operates primarily through MTC tau. The mediation model outputs for Model 4b and Model 4c (where estimated amyloid PiB DVR was the predictor, baseline estimated WMH was the moderator, first available MTC tau was the mediator, and last CDR was the outcome) from the primary model set are presented in Table 4. Table S4 in supporting information includes the mediation model outputs for Model 5b (WMH chronicity as the predictor, baseline amyloid chronicity as the moderator, first available MTC tau as the mediator, and last CDR as the outcome) and Model 5c (estimated WMH as the predictor, baseline estimated amyloid PiB DVR as the moderator, first available MTC tau as the mediator, and last CDR as the outcome) from the secondary model set. Figures S3–S5 in supporting information illustrate the mediation effects of tau pathology for the remaining models. The estimated average causal mediation effects (ACME) were as follows: Model 4c from the primary model set (*β* = 2.133, *p* < 0.001), Model 5b from the secondary model set (*β* = 0.011, *p* = 0.026), and Model 5c from the secondary model set (*β* = 0.019, *p* = 0.006). Bootstrapping confirmed the significance of the indirect effects for all four models, supporting tau as a mechanistic link between amyloid and WMH burden on cognitive decline. The proportion of mediation by tau (prop. mediated) was estimated as follows: Model 4c (primary) 54.3%, Model 5b (secondary) 25.8%, and Model 5c (secondary) 67.6%, indicating that tau accounts for a substantial portion of the observed associations. These findings are consistent with a moderated mediation framework, in which both the indirect and direct pathways vary by WMH burden. This suggests that the contribution of tau and amyloid to cognitive decline may differ across levels of vascular pathology. Figure 4A shows the estimated annual change in MTC SUVR (95% CI) across PiB × WMH chronicity subgroups (< 0, 0–5, > 5 years). When PiB chronicity was < 0 years, estimated annual changes in MTC SUVR were minimal regardless of WMH chronicity. Among individuals with PiB chronicity ≥ 0 years, greater WMH chronicity was associated with larger increases in tau MTC SUVR. This pattern was observed in both the 0 to 5 year and > 5 year PiB chronicity groups, suggesting that the presence of amyloid pathology may amplify the influence of WMH on tau accumulation. The highest estimated annual increase was observed in individuals with both PiB and WMH chronicity > 5 years. Using the moderated mediation model (Model 4b), we estimated predicted annual changes in CDR‐SB across nine PiB × WMH chronicity subgroups. When tau SUVR was set to zero, predicted CDR‐SB changes ranged from 0.19 to 1.46 points per year, with the greatest worsening observed in the PiB > 5 and WMH > 5 group. Including subgroup‐specific median tau SUVR values in the model further increased the predicted rate of CDR‐SB decline when PiB chronicity exceeded 5 years. For example, in the PiB > 5 and WMH > 5 group, the predicted annual CDR‐SB change increased from 1.46 to 3.93 after accounting for tau. The largest increases were observed in subgroups with higher tau burden, supporting tau's role as a mediator in the clinical impact of combined amyloid and WMH chronicity. Figure 4B shows the predicted annual change in CDR‐SB for the same nine subgroups with tau (tau set to the subgroup‐median SUVR) and without tau (tau set to 0). The tau‐related increment (with tau−without tau) was small at lower exposure levels and largest when both PiB and WMH chronicity exceeded 5 years, consistent with a model in which tau increases with joint amyloid–WMH exposure and, in turn, accelerates CDR‐SB worsening. Results were consistent when using Models 4c, 5b, and 5c.

**TABLE 4 alz70831-tbl-0004:** Summary of moderated mediation models (Model 4b and Model 4c).

	MTC			Last CDR‐SB			MTC			Last CDR‐SB		
Predictors	Estimates	CI	*p*	Estimates	CI	*p*	Estimates	CI	*p*	Estimates	CI	*p*
(Intercept)	1.18	1.11–1.25	**<0.001**	0.72	0.29–1.15	**0.001**	1.10	1.05 –1.16	**<0.001**	0.69	0.33 – 1.04	**<0.001**
c65base CDR age	0.00	−0.00−0.00	0.966	0.01	−0.01−0.03	0.354	0.00	−0.00−0.00	0.573	0.01	−0.01 − 0.03	0.284
Female	0.02	−0.03−0.07	0.436	−0.27	−0.55−0.01	0.058	0.03	−0.02−0.07	0.209	−0.27	−0.55 – 0.00	0.050
College	−0.01	−0.05–0.04	0.830	−0.20	−0.48–0.08	0.155	−0.02	−0.06–0.03	0.443	−0.24	−0.51 – 0.04	0.088
Years between MTC and last CDR	−0.01	−0.02to −0.00	**0.031**	0.08	0.02–0.14	**0.012**	−0.01	−0.02 to –0.00	**0.049**	0.08	0.01 – 0.14	**0.017**
PiB chronicity	0.02	0.02–0.03	**<0.001**	0.03	0.01–0.05	**0.009**						
WMH chronicity	0.00	−0.00–0.01	0.382	0.01	−0.0–0.04	0.287						
PiB chronicity ^2	0.00	0.00–0.00	**<0.001**	0.00	0.00–0.00	**0.001**						
PiB chronicity × WMH chronicity	0.00	0.00–0.00	**<0.001**	0.00	0.00–0.00	**0.029**						
WMH chronicity × PiB chronicity^2	0.00	0.00–0.00	**<0.001**	0.00	0.00–0.00	**0.006**						
Estimate amyloid							0.23	0.05–0.41	**0.014**	−1.04	−2.13–0.05	0.061
Estimate WMH							0.00	−0.02–0.01	0.458	0.12	0.04–0.19	**0.002**
Estimate amyloid ^2							0.83	0.56–1.09	**<0.001**	2.89	1.23–4.54	**0.001**
Estimate amyloid × estimate WMH							0.04	0.01–0.07	**0.009**	0.05	−0.13–0.22	0.602
MTC				2.21	1.67–2.75	**<0.001**				2.11	1.58–2.64	**<0.001**
Observations	500			500			500			500		
R^2^ / R^2^ adjusted	0.408 / 0.397			0.301 / 0.287			0.417 / 0.407			0.321 / 0.308		

*Notes*: Regression estimates from moderated mediation models examining how WMH burden moderates the mediated relationship between amyloid, tau pathology, and the final CDR‐SB score. The first two columns show results from the mediation model and the full moderated mediation model for Model 4b, which included amyloid chronicity at the last CDR as the predictor, baseline WMH chronicity as the moderator, and tau SUVR as the mediator. The last two columns present the mediation and full models for Model 4c, using estimated amyloid DVR at the last CDR and estimated baseline WMH values. Both models include covariates for sex, education, and the age difference between tau PET and the last CDR assessment. Bolded values indicate statistically significant predictors (*p* < 0.05).

Abbreviations: Abbreviations: AICc, Akaike information criterion‐corrected; CDR, Clinical Dementia Rating; CDR‐SB, Clinical Dementia Rating‐Sum of Boxes; DVR, distribution volume ratio; ICC, intraclass correlation; IQR, interquartile range; MTC, meta‐temporal composites; PiB, Pittsburgh compound B; SUVR, standardized uptake value ratio; WMH, white matter hyperintensities.

Similarly, exploratory analysis showed that tau pathology significantly mediated the synergistic effect of WMH and amyloid on annualized CDR‐SB change. The annualized CDR change was larger in the WMH+ and PiB+ subsets and was highest in the WMH+PiB+ subset, as shown in Figure S6 in supporting information. The mean (SD) estimated WMH at tau baseline was 2.06 (4.2). In the WMHV+ subset (*n* = 111, 22.2%), the mean (SD) WMH chronicity was 4.93 (7.4). The mean (SD) estimated amyloid at tau baseline was 1.18 (0.2). In the PiB+ subset (*n* = 165, 33%), the mean (SD) amyloid chronicity was 7.48 (7.2). The mediation model outputs for Model 6b and Model 6c from the primary model set are presented in Table S5 in supporting information, while Table S6 in supporting information contains the mediation model outputs for Model 7b and Model 7c from the secondary model set. Figures S7–S10 in supporting information illustrate the mediation effects of tau pathology across all models, demonstrating tau's contribution to the association between WMH, amyloid chronicity, estimated WMH, amyloid at baseline tau, and annualized CDR change in both primary and secondary model sets, except for Model 7c in the secondary model set. The estimated ACME values were as follows: Primary model set—Model 6b (*β* = 0.002, *p* < 0.001), Model 6c (*β* = 0.188, *p* < 0.001); Secondary model set Model 7b (*β* = 0.0005, *p* = 0.040). The ACME was not significant in Model 7c (*β* = 0.0001, *p* = 0.45) from the secondary model set, but MTC moderated the relationship between estimated amyloid at tau and annualized CDR change.[Table alz70831-tbl-0004], [Fig alz70831-fig-0004]

## 4 DISCUSSION

Expanding the application of A and V biomarkers to unimpaired individuals before cognitive and functional symptoms could potentially offer an opportune window for implementing prevention strategies for these diseases and provide an opportunity for prognostic information, such as estimated years to symptoms, to inform treatment and life planning decisions. This study examined the interplay between amyloid and cerebrovascular burden (as indexed by WMH) in relation to cognitive decline and the mediating role of tau pathology. We operationalized biomarker burden in two related ways. First, we operationalized burden in terms of its estimated chronicity, or the number of years since having passed a threshold of biomarker positivity. Using that operationalization, we found that both amyloid and WMH chronicity contributed to cognitive decline, with greater WMH burden exacerbating amyloid‐related cognitive deterioration. Results were consistent when using estimated PiB amyloid or WMH volumes. Additionally, tau pathology emerged as a key mediator in the association between amyloid and cerebrovascular burden (WMH) and cognitive impairment, suggesting that tau accumulation may represent a downstream pathway through which these upstream pathologies contribute to cognitive decline. These findings highlight the importance of considering coexisting vascular and neurodegenerative pathologies in the progression of cognitive decline and reinforce the need for temporal biomarker models to refine prognostic estimates in individuals at risk for AD.

To maximize completeness and minimize selection bias, we reported SBP, DBP, BMI, total cholesterol, and creatinine, which had high coverage in our cohort. Given heterogeneity in MRI sequences, segmentation pipelines, and scaling across studies, we emphasized within‐cohort interpretation of WMH using distributions and a prespecified positivity threshold rather than numeric cross‐cohort comparisons. At a mean baseline age of 62.6 years, 66.8% had any adjusted WMH; in the literature, any WMH is commonly observed in roughly 20% to 50% by midlife and in > 90% at advanced ages.^34^

Extending SILA to WMH is appropriate because WMH increases with age at the group level, and a time‐above‐threshold metric aids clinical interpretation. Although WMH is more sensitive than amyloid DVR to segmentation and scanner differences, harmonized preprocessing and QC produced stable inferences. We used the validated WMH‐SILA framework and retained the previously published 2.06 mL threshold to promote comparability.^10^ Alternative plausible thresholds would translate the chronicity scale but are unlikely to change qualitative associations, provided the cut‐point remains within a clinically defensible range. For broader clinical use, harmonized scanner‐aware thresholds and shared reference standards will be important.

Our findings support the role of cerebrovascular pathology as a modifier of the clinical course in individuals with underlying AD pathology, demonstrating that the presence of amyloid accumulation and greater WMH burden is associated with faster cognitive decline. Prior studies have reported similar findings: higher volumes of WMH were associated with a shorter interval between amyloid onset and cognitive impairment,^22^ and WMH burden has been linked to increased dementia risk and accelerated neurodegeneration, even independent of amyloid pathology.^11^ Additionally, research suggests that vascular comorbidities interact with amyloid to amplify neurodegeneration and clinical impairment.^35^, ^36^, ^37^, ^38^ Our study extends this literature by incorporating a chronicity‐based approach and estimating biomarker values at the same time points for both amyloid and vascular pathology, demonstrating that higher WMH burden, such as longer WMH duration or greater WMH volume, plays a critical role in accelerating amyloid‐related cognitive decline. Furthermore, our model‐implied translation of predicted slopes indicates that the years required to move from a CDR‐SB of 0.5 to 6.5 decrease as WMH chronicity increases, holding amyloid chronicity within a given stratum constant (see section 2.6.1). These findings illustrate how coexisting vascular pathology may accelerate amyloid‐associated clinical progression and reinforce the hypothesis that WMH burden modifies the clinical impact of AD pathology. These results are consistent with previous findings^17^, ^18^ that individuals who became amyloid positive at an older age tend to have shorter intervals between amyloid onset and symptom emergence.

Level‐based specifications (Models 1c, 2c) showed better in‐sample fit than chronicity‐based specifications (Models 1b, 2b), which is plausible because current levels retain the magnitude of pathology at each assessment and can track near‐term change, whereas chronicity captures exposure time and is valuable for staging and prognosis. Because substantive patterns, including the amyloid × WMH interaction and tau results, were consistent across parameterizations, we report both to balance clinical interpretability and statistical fit. Within the best‐fitting specification, block‐wise tests and permutation importance indicated that amyloid terms accounted for a larger share of explained variance in CDR‐SB change than WMH terms, while WMH remained a significant modifier of amyloid‐related decline. This supports a framework in which amyloid burden primarily drives slope differences and WMH amplifies that effect.

Baseline age did not reach significance once time‐varying biomarker burden (chronicity or level) and random intercepts were included. This pattern is consistent with shared variance between age and pathology, the cohort's baseline age distribution (mean 62.63, SD 7.24), and centering at 65, all of which reduce residual age‐related signal. Within this sample, biomarker burden, rather than chronological age, primarily accounts for variation in CDR‐SB trajectories.

The results from Aim 2 suggest that tau pathology mediates the association between amyloid chronicity and cognitive decline, with WMH burden modifying this relationship. We observed a synergistic effect of amyloid and WMH chronicity on tau accumulation in the MTC: WMH chronicity had minimal impact in amyloid‐negative individuals, but among those with PiB chronicity ≥ 0 years, greater WMH chronicity was associated with steeper annual increases in MTC SUVR. These results support prior work suggesting that vascular pathology can amplify tau accumulation in the presence of amyloid (Rabin et al.^39^). Including tau SUVR in the model increased the estimated rate of CDR‐SB worsening, particularly in subgroups with high PiB and WMH chronicity, indicating that tau accumulation may account for a substantial portion of the cognitive impact of coexisting amyloid and vascular pathology. These findings support the hypothesis that amyloid and WMH contribute to downstream tau pathology, which in turn drives cognitive deterioration.^39^, ^40^ They also underscore the utility of tau PET imaging for risk stratification and suggest that tau‐targeted interventions may be especially relevant in individuals with mixed pathology profiles. Our results align with previous studies showing that tau is a stronger predictor of cognitive decline than amyloid alone,^14^, ^41^, ^42^, ^43^, ^44^ and that tau accumulation is tightly linked to neurodegeneration.^45^, ^46^, ^47^ Additionally, our cross‐sectional mediation analysis suggests that WMH burden may indirectly contribute to tau accumulation, consistent with literature implicating cerebrovascular dysfunction in tauopathy via mechanisms such as blood–brain barrier breakdown and neuroinflammation.^37^, ^48^, ^49^ Although tau PET was available only cross‐sectionally, these findings highlight the importance of considering vascular markers as contributors to tau‐mediated cognitive decline. Future work incorporating longitudinal tau imaging and extended cognitive follow‐up is needed to further clarify these interactions and to examine resilience factors, such as lifestyle and health behaviors, that may modulate the trajectory of AD.^50^ Compared to prior studies, our chronicity‐based approach offers a more temporally nuanced view of how amyloid, WMH, and tau jointly shape clinical progression.

Study limitations include the following. We characterized increasing impairment using the CDR, but this may be too coarse an instrument to identify more subtle symptoms of cognitive decline. Future work could examine cognitive change in specific cognitive domains such as episodic memory and executive function, which have shown early changes in prior studies.^12^, ^51^, ^52^ Observations reported here with PiB and MK‐6240 tau need to be replicated with other amyloid PET and tau PET tracers and fluid biomarkers. Operationalizing cerebrovascular disease (V) using WMH alone, while common in aging studies, may not fully capture the complexity of small vessel disease. Future work incorporating diffusion imaging may help detect earlier white matter changes and clarify more nuanced links between vascular pathology and amyloid accumulation. Additionally, we have only included sex and education as covariates, but we know from other work in our group that sex differences may alter the amyloid–tau timeline.^53^ Future analyses should examine the moderation or mediation effect of these covariates and other key variables, for example, apolipoprotein E ε4 carrier status. Another limitation concerns estimating WMH when the modeled rate of change is near zero; in such flat regions, temporal fits can diverge or converge to local minima. Future studies will consider model constraints, alternative parameterizations, or more robust optimization techniques to mitigate this issue. In addition, these results, which were obtained on a convenience sample, may differ in other populations and cohorts. The limited ethnocultural diversity in the sample limits the generalizability of the findings, and efforts are under way to increase representation in biomarker research.

## 5 CONCLUSIONS

Overall, our findings emphasize the significance of amyloid chronicity in understanding cognitive decline trajectories and underscore the critical role of cerebrovascular pathology in modifying disease progression. The significant mediation by tau suggests that targeting tau accumulation in addition to addressing amyloid and vascular health may be a promising therapeutic approach to mitigate cognitive deterioration. Temporal biomarker modeling, such as the methods used in this study, will be instrumental in identifying individuals at risk for accelerated cognitive decline and refining intervention strategies in future research and clinical applications.

## CONFLICT OF INTEREST STATEMENT

S.C.J. has served as a consultant to Enigma Biomedical, Eli Lilly, and AlzPath. The remaining authors have no relevant disclosures. All author disclosures are in the supporting information.

## CONSENT STATEMENT

The study procedures received approval from the University of Wisconsin–Madison Institutional Review Board and were conducted in compliance with the World Medical Association Declaration of Helsinki. All subjects provided informed consent.

## Supporting information



Supporting Information

Supporting Information
