# Supplementary material for: Tau mediates the impact of amyloid and vascular disease burden on the trajectory of clinical symptoms
Source: Alzheimers Dement. 2025 Oct 30;21(10):e70831. doi: 10.1002/alz.70831 (PMC12572831; doi:10.1002/alz.70831)
Supplement: Supplementary file 1 — Supporting Information [file ALZ-21-e70831-s002.docx]

**FIGURE CAPTIONS**

**Supplementary Figure 1. Participant Inclusion Flow Chart.** Flow chart illustrating the selection of participants included in Aim 1 amd Aim 2 analyses. WRAP and WADRC cohort contributions are also indicated.

**Supplementary Figure 2. Spaghetti plots of CDR-Sum of Boxes trajectories across different time scales by baseline PiB/WMH status.** Spaghetti plots showing individual-level CDR-Sum of Boxes (CDR-SB) trajectories across five different time scales, corresponding to the models in Aim 1 (Models 1b, 1c, 2b, 2c, and 3), stratified by PiB/WMH status at baseline. PiB and WMH statuses were determined using estimated amyloid DVR and WMH values at the first CDR assessment via the SILA method.Top panel: Left—CDR-SB vs. amyloid chronicity (Model 1b); Right—CDR-SB vs. estimated amyloid DVR (Model 1c); Middle panel: Left—CDR-SB vs. WMH chronicity (Model 2b); Right—CDR-SB vs. estimated WMH volume (Model 2c); Bottom panel: CDR-SB vs. age (Model 3). Colored lines and points represent individual trajectories by PiB/WMH status. Blue curves represent fitted loss functions across each time scale. Shaded background regions indicate dementia severity categories based on CDR-SB thresholds from O’Bryant et al. (2010): 2.5–4.0 (very mild), 4.5–9.0 (mild), 9.5–15.5 (moderate), and 16.0–18.0 (severe).

**Supplementary Figure 3. Moderated mediation analysis of last observed CDR-SB (Model 4c).** Meta-temporal tau levels mediate the synergistic effect of estimated WMH and amyloid DVR on last CDR‐SB. A. We modeled amyloid DVR at last CDR as predictor, baseline WMH as moderator, first available MTC tau as mediator, and last CDR as outcome, adjusting for baseline age, sex, education, and time between study baseline and first tau PET. Results indicate that MTC tau significantly mediated the effect of amyloid DVR on the final CDR-SB score in individuals with low (Figure B, left panel) and high WMH estimated values (Figure B, right panel), accounting for 54.3% of the total effects. A statistically significant direct effect was observed in low WMH values. Tau was measured with florquinitau PET(MK6240; Meta-temporal (MTC) SUVR composites derived from 70-90 min post-injection). ACME = Average Causal Mediation Effects, ADE = Average Direct Effect.

**Supplementary Figure 4. Moderated mediation analysis of last observed CDR-SB (Model 5b).** Meta-temporal tau levels mediate the synergistic effect of amyloid chronicity and WMH chronicity on last CDR‐SB. A. We modeled WMH chronicity as predictor, baseline amyloid chronicity as moderator, first available MTC tau as mediator, and last CDR as outcome, adjusting for baseline age, sex, education, and time between study baseline and first tau PET. Results indicate that MTC tau significantly mediated the effect of WMH chronicity on CDR-SB in individuals with low (Figure B, left panel) and high amyloid chronicity (Figure B, right panel), accounting for 25.8% of the total effects. No statistically significant direct effect and total effect were observed. Tau was measured with florquinitau PET(MK6240; Meta-temporal (MTC) SUVR composites derived from 70-90 min post-injection). ACME = Average Causal Mediation Effects, ADE = Average Direct Effect.

**Supplementary Figure 5. Moderated mediation analysis of last observed CDR-SB (Model 5c).** Meta-temporal tau levels mediate the synergistic effect of amyloid DVR and WMH on last CDR‐SB. A. We modeled WMH as predictor, baseline amyloid DVR as moderator, first available MTC tau as mediator, and last CDR as outcome, adjusting for baseline age, sex, education, and time between study baseline and first tau PET. Results indicate that MTC tau significantly mediated the effect of WMH on CDR-SB in individuals with low (Figure B, left panel) and high amyloid DVR (Figure B, right panel), accounting for 67.6% of the total effects. No statistically significant direct effect and total effect were observed. Tau was measured with florquinitau PET(MK6240; Meta-temporal (MTC) SUVR composites derived from 70-90 min post-injection). ACME = Average Causal Mediation Effects, ADE = Average Direct Effect.

**Supplementary Figure 6. Bubble plots of annualized CDR-SB change by PiB/WMH status at last assessment.** Bubble plots illustrating annualized change in CDR-Sum of Boxes (CDR-SB) by PiB/WMH status at the time of last available PiB and WMH measurements.Top panel: Amyloid chronicity (x-axis) vs. WMH chronicity (y-axis); Bottom panel: Estimated PiB DVR at CDR (x-axis) vs. Estimated WMH volume at CDR (y-axis). Each circle represents an individual, with the size of the circle proportional to their annualized CDR-SB change. Colors indicate PiB/WMH groupings, defined by biomarker positivity at the last CDR assessment.

**Supplementary Figure 7. Moderated mediation analysis of annualized CDR-SB score change (Model 6b).** Meta-temporal tau levels mediate the synergistic effect of WMH chronicity and amyloid chronicity on annualized CDR-SB score change. A. We modeled amyloid chronicity at tau as predictor, WMH chronicity at tau as moderator, first available MTC tau as mediator, and annualized CDR-SB score change as outcome, adjusting for baseline age, sex and education. Results indicate that MTC tau significantly mediated the effect of amyloid chronicity on annualized CDR-SB score change in individuals with low (Figure B, left panel) and high WMH chronicities (Figure B, right panel). No statistically significant direct effect was observed. Tau was measured with florquinitau PET(MK6240; Meta-temporal (MTC) SUVR composites derived from 70-90 min post-injection). ACME = Average Causal Mediation Effects, ADE = Average Direct Effect.

**Supplementary Figure 8. Moderated mediation analysis of annualized CDR-SB score change (Model 6c).** Meta-temporal tau levels mediate the synergistic effect of estimate WMH and amyloid DVR on annualized CDR-SB score change. A. We modeled amyloid DVR at tau as predictor, WMH at tau as moderator, first available MTC tau as mediator, and annualized CDR-SB score change as outcome, adjusting for baseline age, sex, and education. Results indicate that MTC tau significantly mediated the effect of amyloid DVR on annualized CDR-SB score change in individuals with low (Figure B, left panel) and high WMH estimated values (Figure B, right panel). No statistically significant direct effect was observed. Tau was measured with florquinitau PET(MK6240; Meta-temporal (MTC) SUVR composites derived from 70-90 min post-injection). ACME = Average Causal Mediation Effects, ADE = Average Direct Effect.

**Supplementary Figure 9. Moderated mediation analysis of annualized CDR-SB score change (Model 7b).** Meta-temporal tau levels mediate the synergistic effect of amyloid chronicity and WMH chronicity on annualized CDR-SB score change. A. We modeled WMH chronicity at tau as predictor, amyloid chronicity at tau as moderator, first available MTC tau as mediator, and annualized CDR-SB score change as outcome, adjusting for baseline age, sex, education. Results indicate that MTC tau significantly mediated the effect of WMH chronicity on annualized CDR-SB score change in individuals with low (Figure B, left panel) and high amyloid chronicity (Figure B, right panel). No statistically significant direct effect and total effect were observed. Tau was measured with florquinitau PET(MK6240; Meta-temporal (MTC) SUVR composites derived from 70-90 min post-injection). ACME = Average Causal Mediation Effects, ADE = Average Direct Effect.

**Supplementary Figure 10. Moderated mediation analysis of annualized CDR-SB score change (Model 7c).** Meta-temporal tau levels mediate the synergistic effect of amyloid DVR and WMH on annualized CDR-SB score change. A. We modeled WMH at tau as predictor, amyloid DVR at tau as moderator, first available MTC tau as mediator, and annualized CDR-SB score change as outcome, adjusting for baseline age, sex and education. Results indicate that MTC tau significantly mediated the effect of WMH on annualized CDR-SB score change in individuals with low (Figure B, left panel) and high amyloid DVR (Figure B, right panel). No statistically significant direct effect and total effect were observed. Tau was measured with florquinitau PET(MK6240; Meta-temporal (MTC) SUVR composites derived from 70-90 min post-injection). ACME = Average Causal Mediation Effects, ADE = Average Direct Effect.


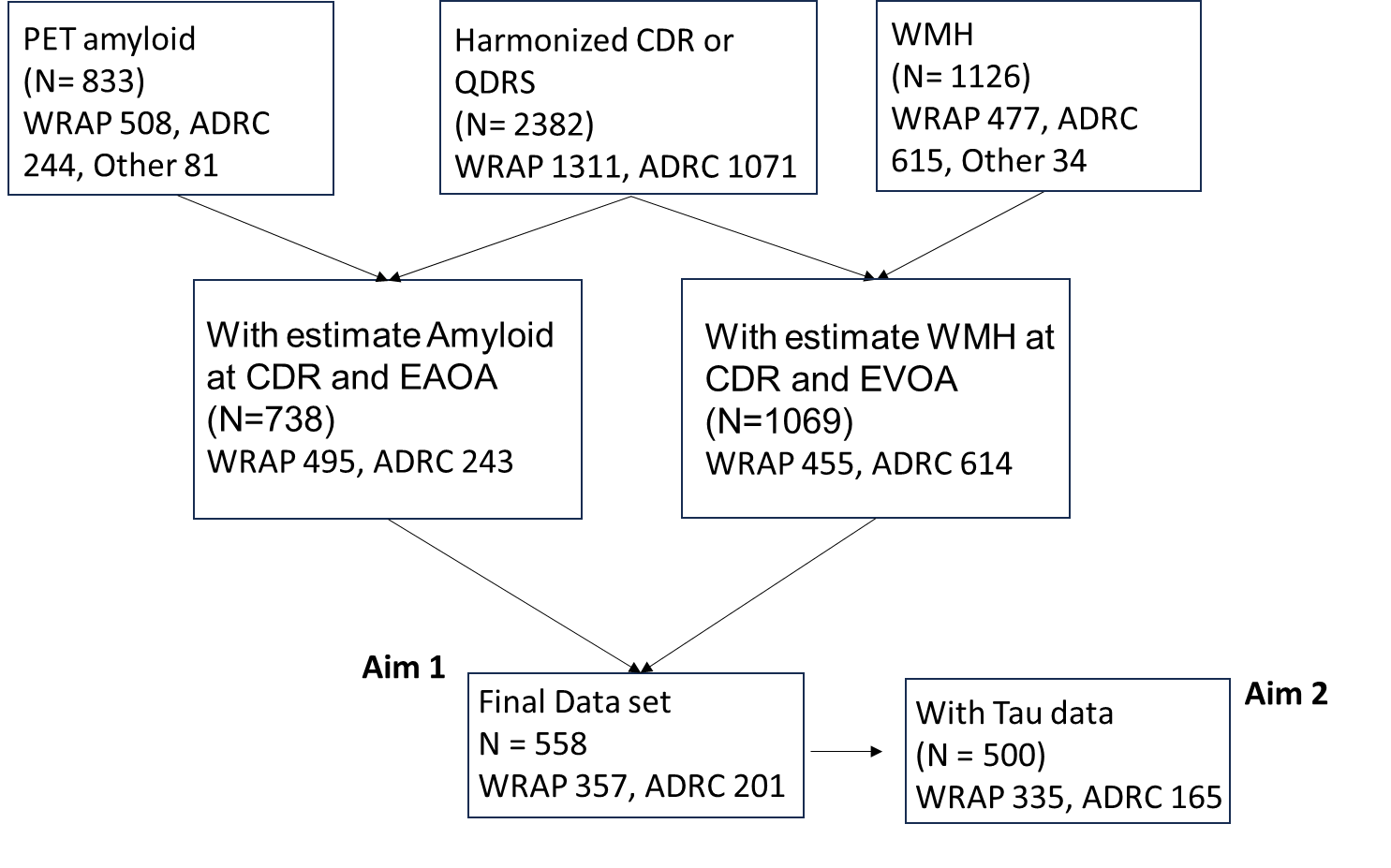


**Supplementary Figure 1. Participant Inclusion Flow Chart.**


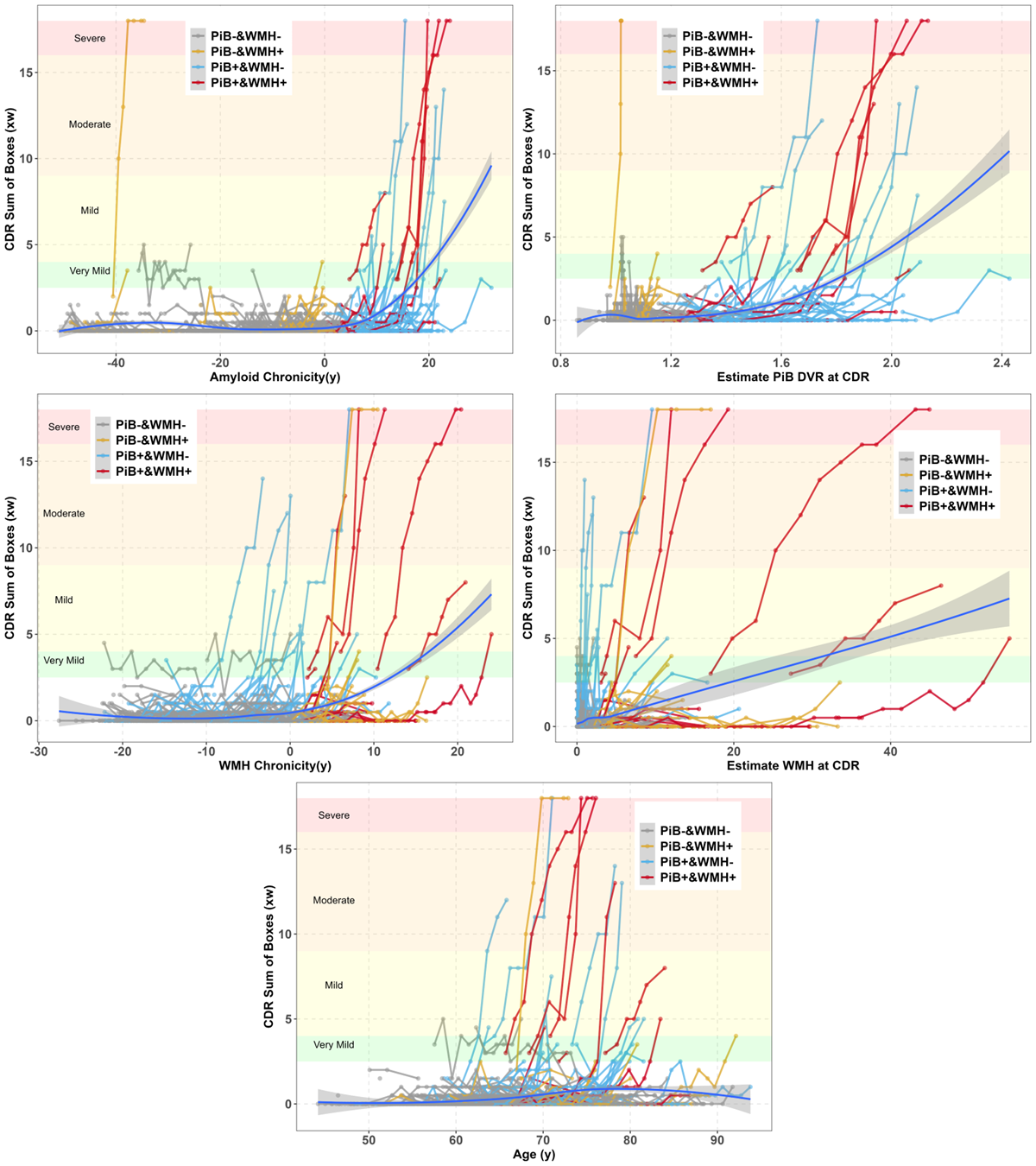


**Supplementary Figure 2. Spaghetti plots of CDR-Sum of Boxes trajectories across different time scales by baseline PiB/WMH status.**


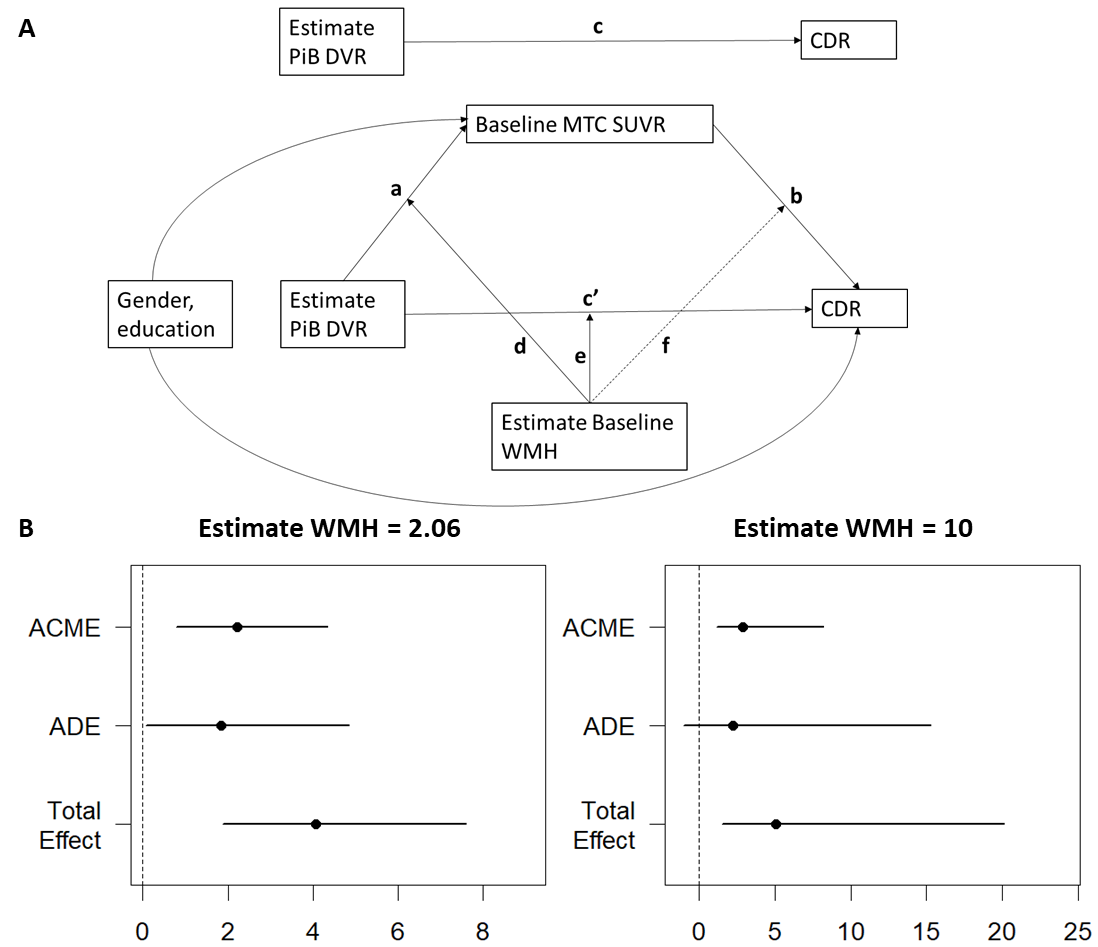


**Supplementary Figure 3. Moderated mediation analysis of last observed CDR-SB (Model 4c**).


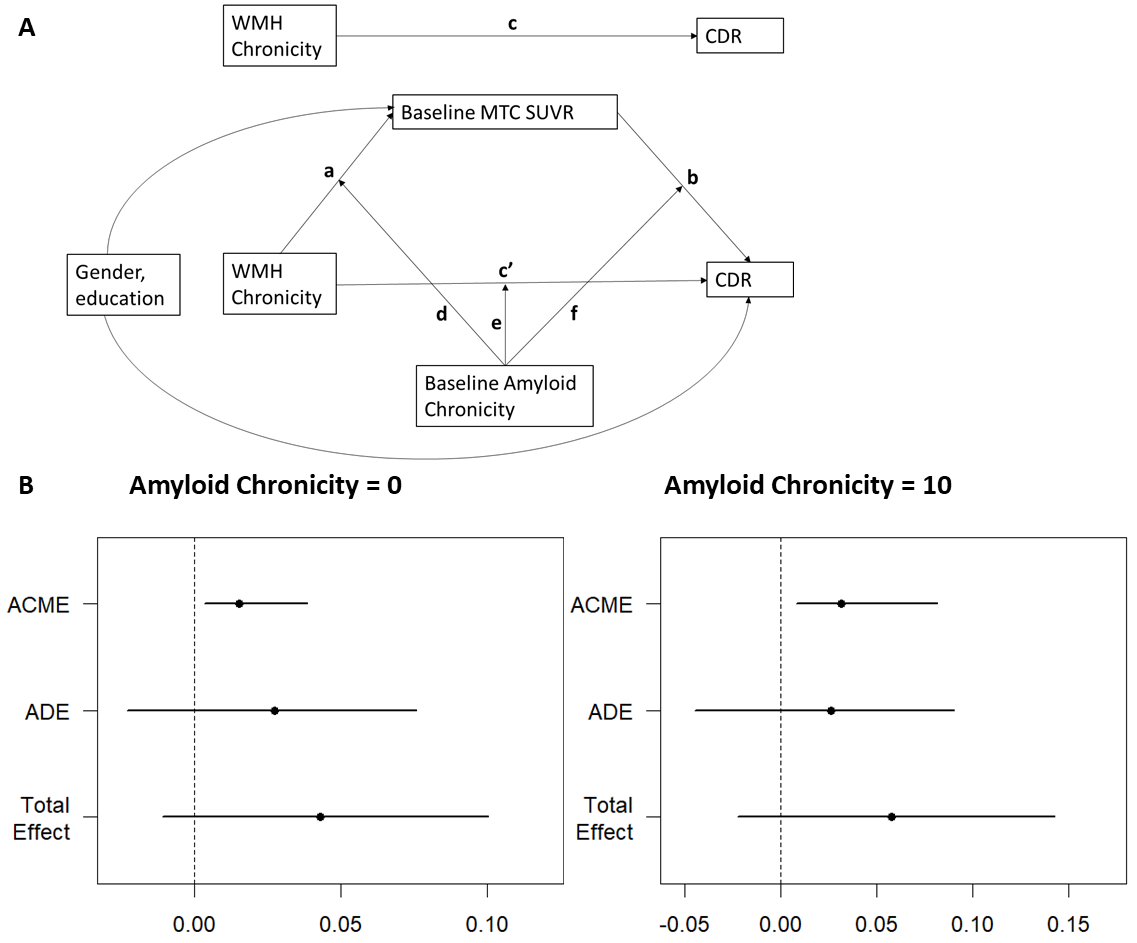


**Supplementary Figure 4.** **Moderated mediation analysis of last observed CDR-SB (Model 5b**).


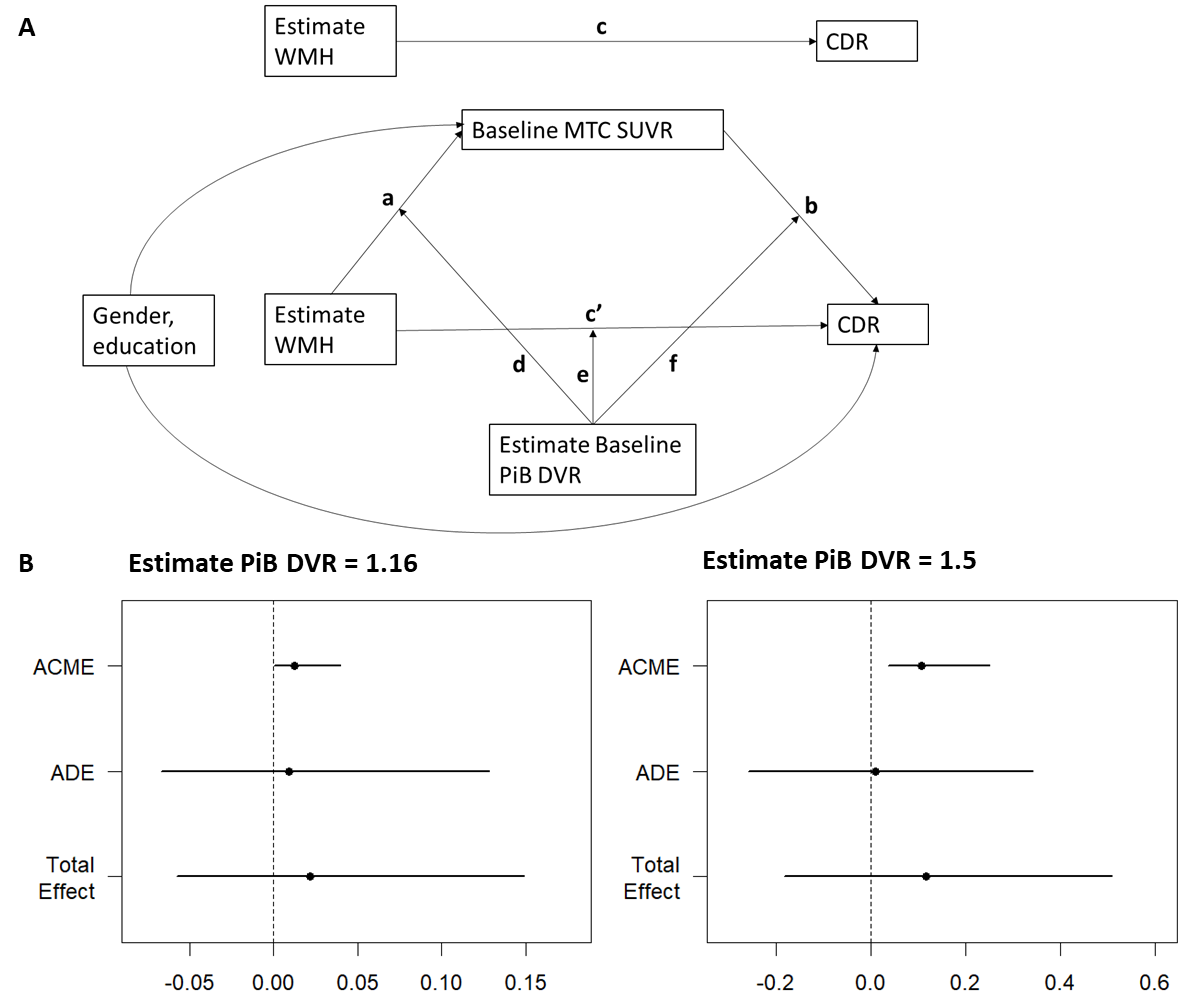


**Supplementary Figure 5. Moderated mediation analysis of last observed CDR-SB (Model 5c**).


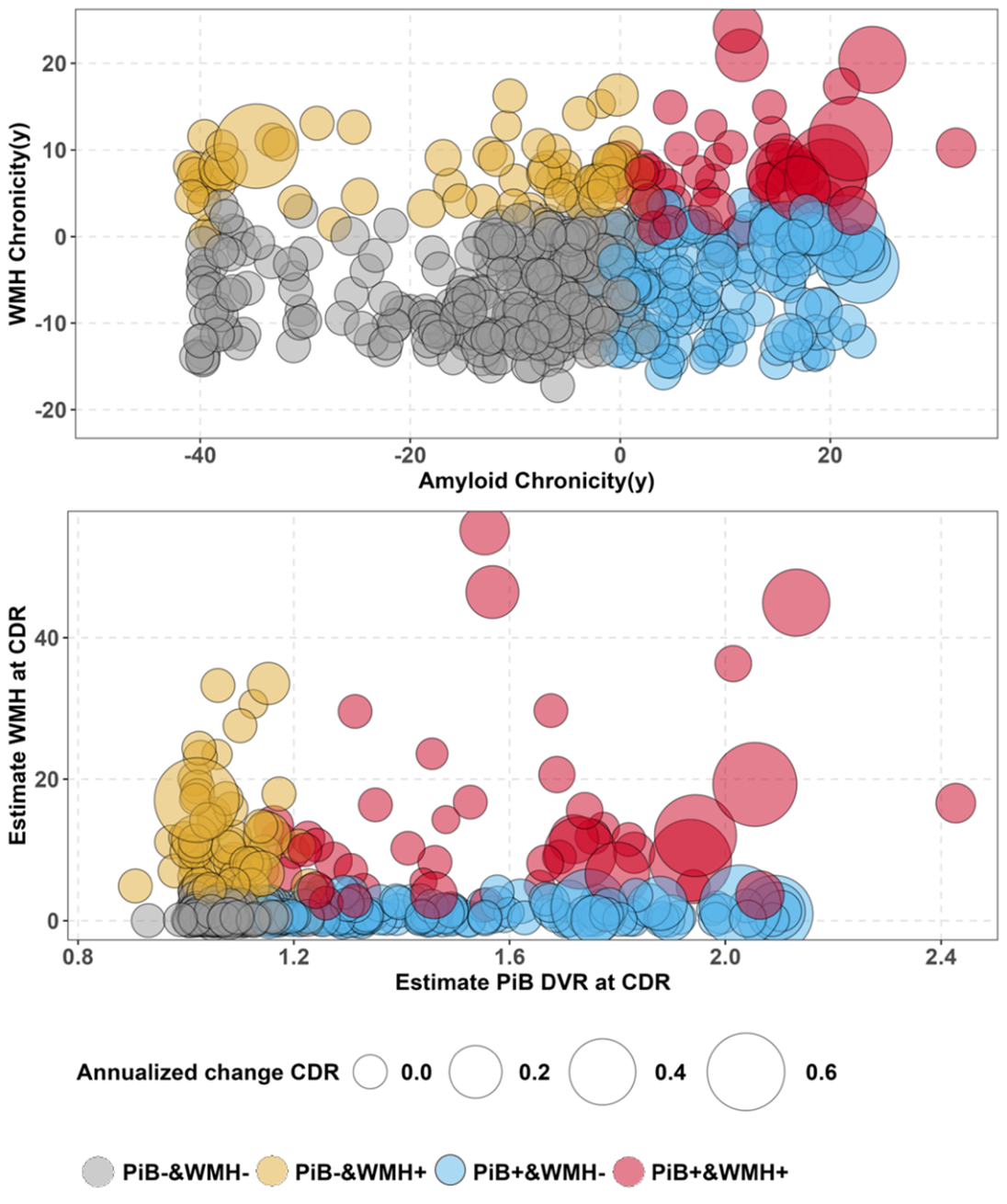


**Supplementary Figure 6. Bubble plots of annualized CDR-SB change by PiB/WMH status at last assessment.**


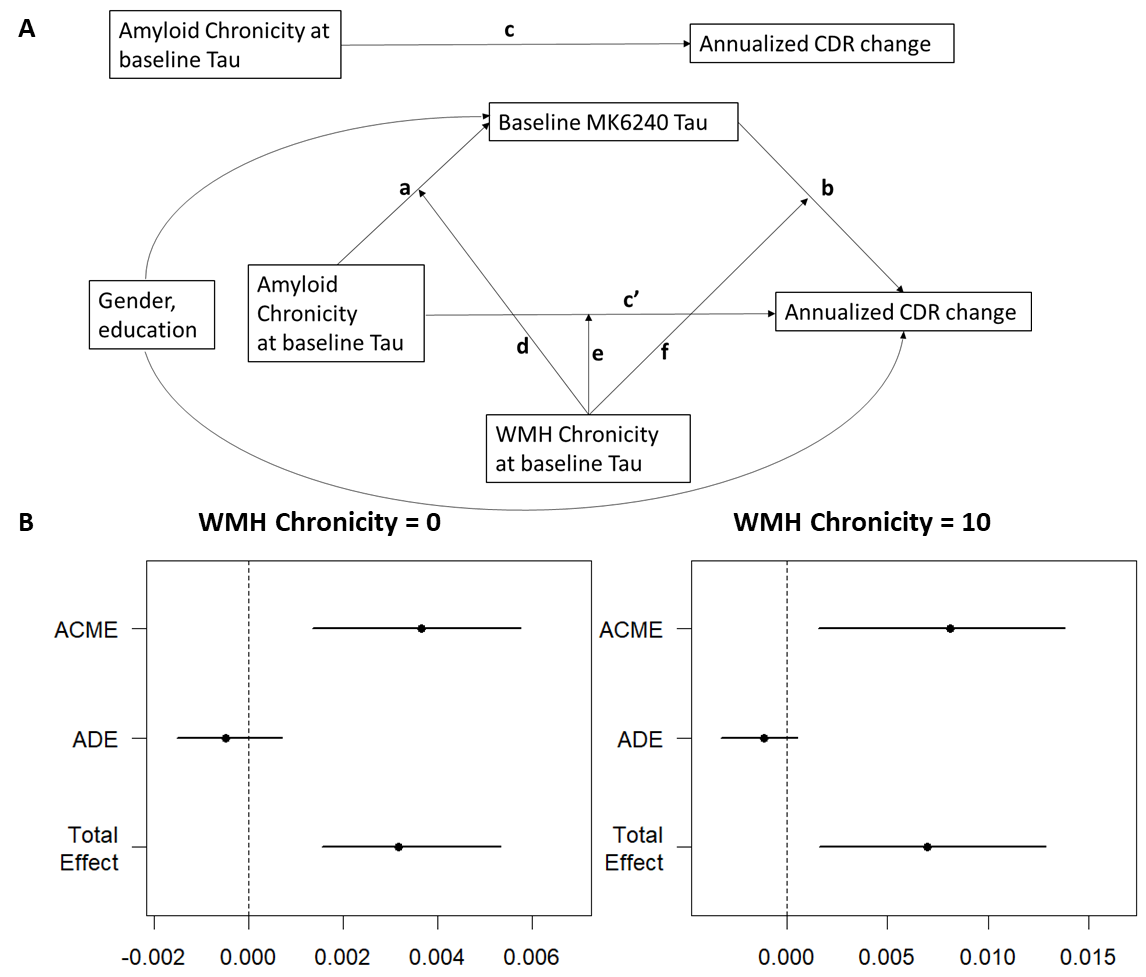


**Supplementary Figure 7. Moderated mediation analysis of annualized CDR-SB score change (Model 6b).**


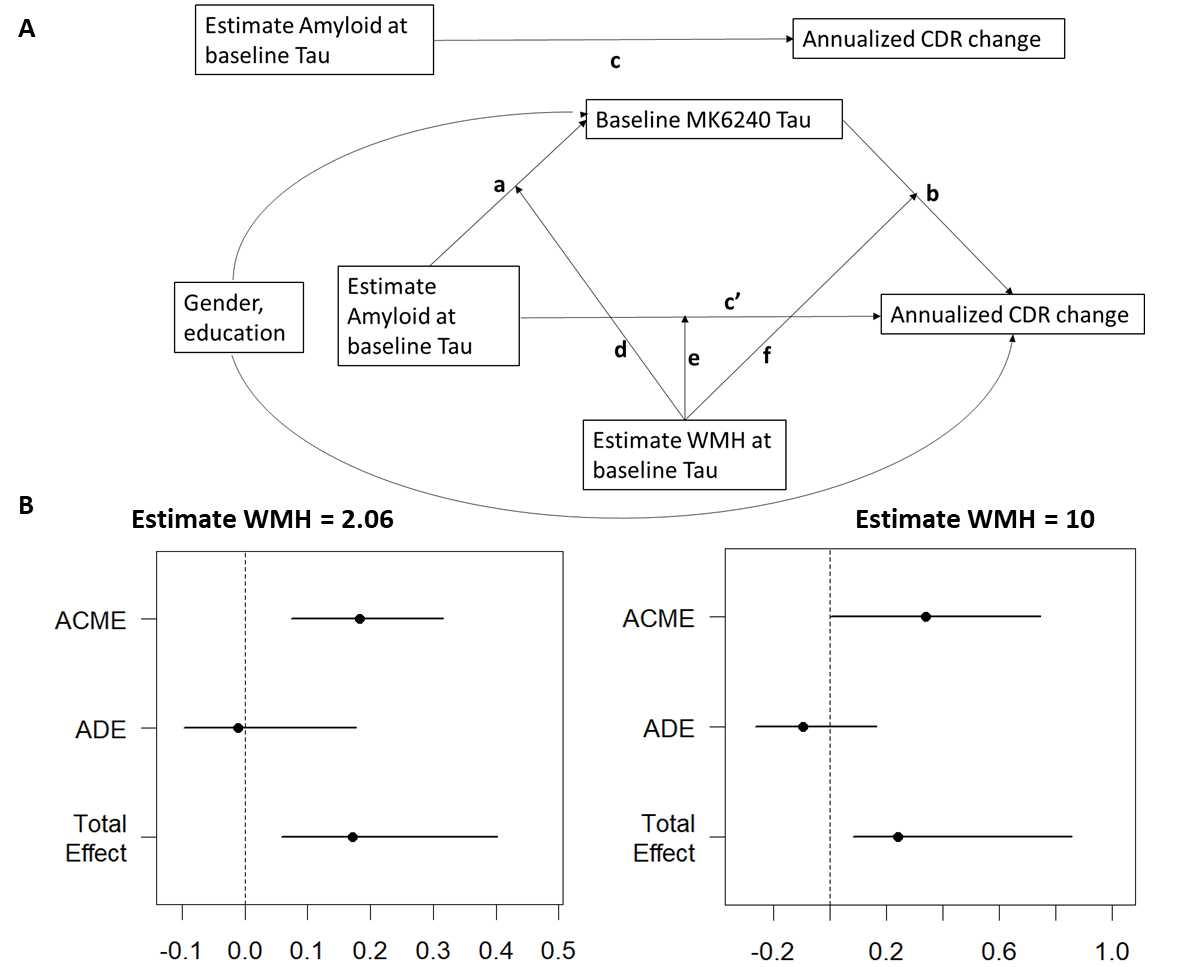


**Supplementary Figure 8. Moderated mediation analysis of annualized CDR-SB score change (Model 6c).**


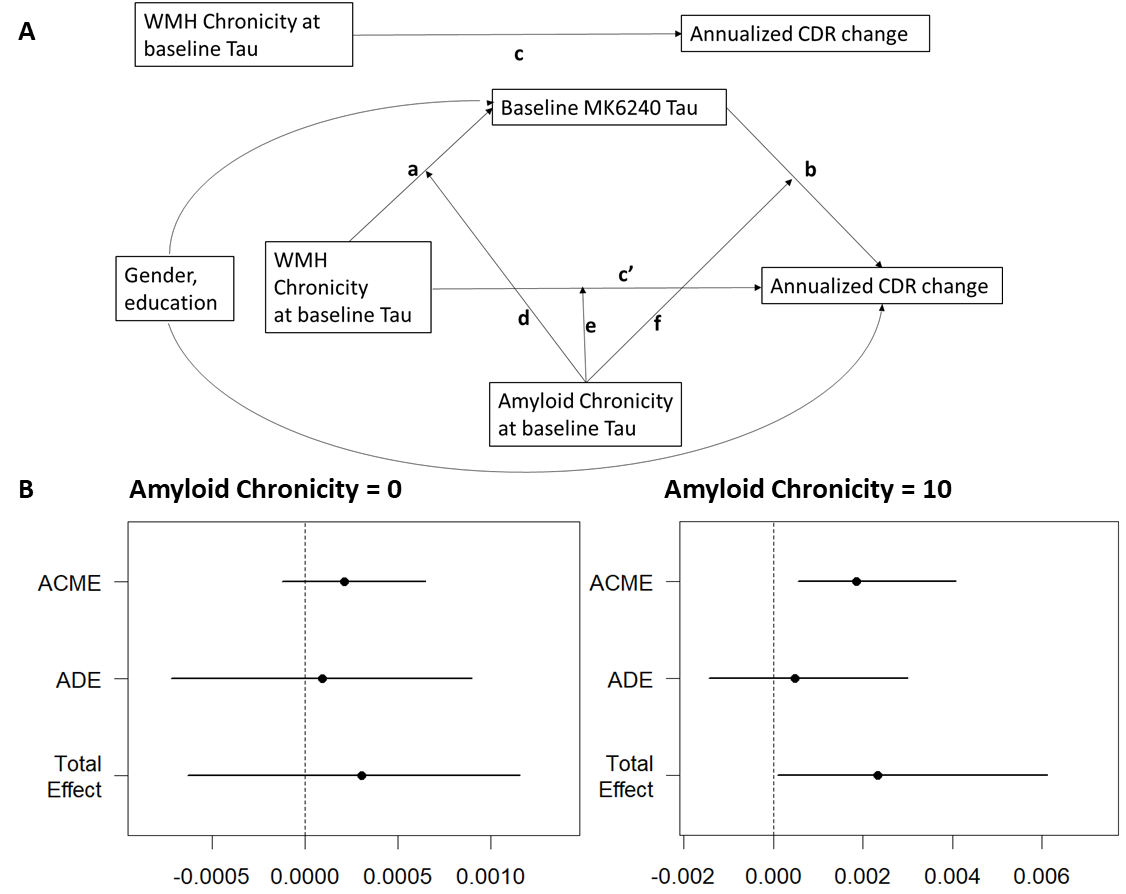


**Supplementary Figure 9. Moderated mediation analysis of annualized CDR-SB score change (Model 7b).**


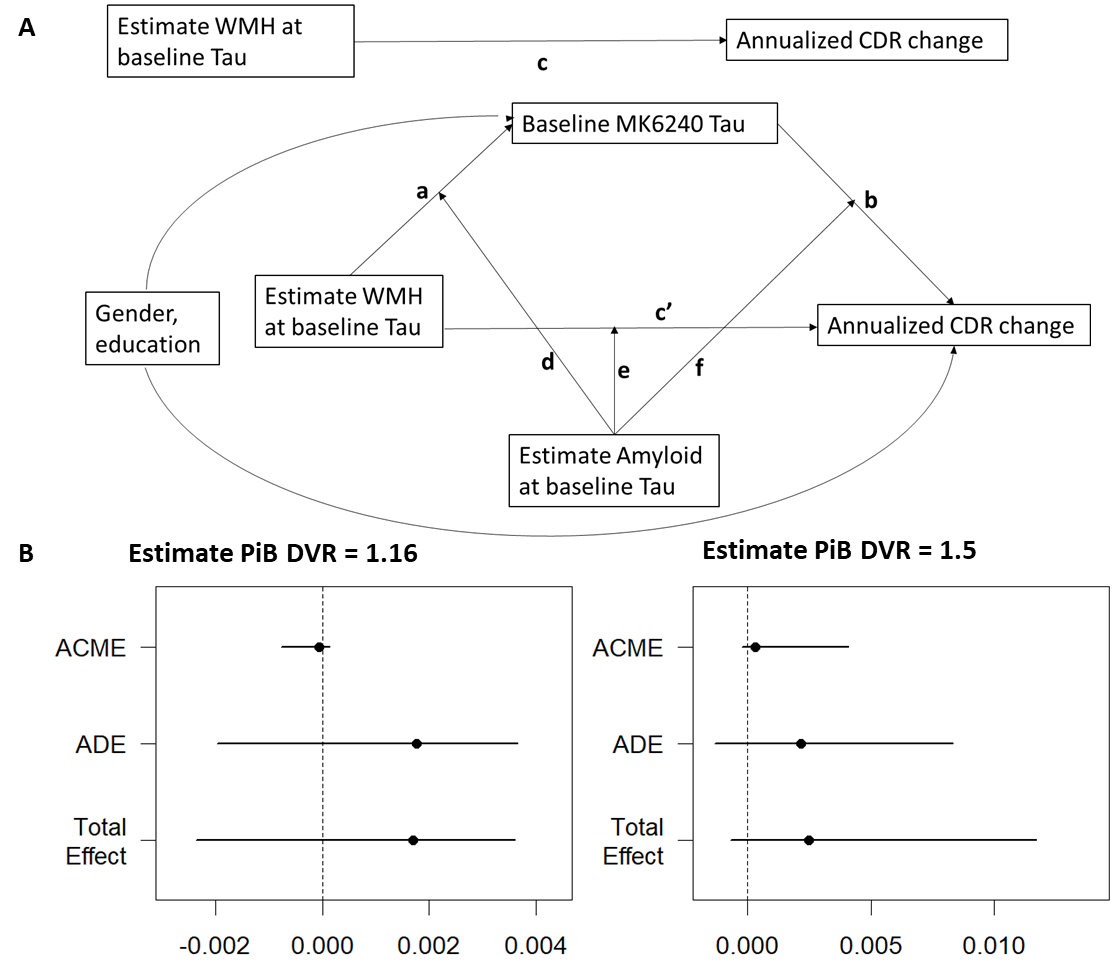


**Supplementary Figure 10. Moderated mediation analysis of annualized CDR-SB score change (Model 7c).**

Supplementary Table 1 Mixed effect model output (Age as timescale)

|  | **Model 3** | | |
| --- | --- | --- | --- |
| *Predictors* | *Estimates* | *CI* | *p* |
| (Intercept) | 0.40 | 0.14 – 0.67 | **0.003** |
| CDR age (center at 65) | -0.00 | -0.01 – 0.01 | 0.908 |
| PiB-WMH+ | 0.13 | -0.38 – 0.64 | 0.612 |
| PiB+WMH- | 0.20 | -0.12 – 0.52 | 0.221 |
| PiB+WMH+ | -1.28 | -2.02 – -0.55 | **0.001** |
| Female | -0.20 | -0.43 – 0.03 | 0.087 |
| College | -0.21 | -0.44 – 0.02 | 0.076 |
| CDR age × PiB-WMH+ | 0.06 | 0.02 – 0.10 | **0.004** |
| CDR age × PiB+WMH- | 0.10 | 0.07 – 0.12 | **<0.001** |
| CDR age × PiB+WMH+ | 0.50 | 0.44 – 0.56 | **<0.001** |
| **Random Effects** | | | |
| σ^2^ | 0.73 | | |
| τ_00_ _reggieid_ | 1.42 | | |
| ICC | 0.66 | | |
| N _reggieid_ | 558 | | |
| Observations | 2567 | | |
| Marginal R^2^ / Conditional R^2^ | 0.272 / 0.752 | | |
| AICc | 7709.97 | | |

Note: Model 3: CDR SoB= Time varying CDR age (and quadratic if sig)+ PiB/WMH status at baseline CDR (based on estimated values using SILA method (<https://github.com/Betthauser-Neuro-Lab/SILA-AD-Biomarker)>) + interaction + covariates (gender, education) + random effects

**Supplementary Table 2 Block-wise contribution within Model 1c (ML fit).**

| Comparison | LRT $\boldsymbol{\chi}^{\boldsymbol{2}}$ | p-value | AICc (full) | AICc (reduced) | ΔAICc | $\boldsymbol{R}_{\boldsymbol{marg}}^{\boldsymbol{2}}$(full) | $\boldsymbol{R}_{\boldsymbol{marg}}^{\boldsymbol{2}}$(reduced) | $\boldsymbol{\Delta}\boldsymbol{R}_{\boldsymbol{marg}}^{\boldsymbol{2}}$ |
| --- | --- | --- | --- | --- | --- | --- | --- | --- |
| Full 1c vs. 1c minus {amyloid block = {$\boldsymbol{PiB}$, $\boldsymbol{PiB}^{\boldsymbol{2}}$, $\boldsymbol{PiB}\boldsymbol{\times WMH}$, $\boldsymbol{PiB}^{\boldsymbol{2}}\boldsymbol{\times WMH}$ }} | 738.65 | <.001 | 7312.31 | 8042.90 | 730.59 | 0.400 | 0.102 | 0.300 |
| Full 1c vs. 1c minus {WMH block = {$\boldsymbol{WMH}$, $\boldsymbol{PiB}\boldsymbol{\times WMH}$, $\boldsymbol{PiB}^{\boldsymbol{2}}\boldsymbol{\times WMH}$ }} | 276.28 | <.001 | 7312.31 | 7582.54 | 270.23 | 0.400 | 0.251 | 0.151 |

Note: Larger LRT, ΔAICc, and $\Delta R_{marg}^{2}$ indicate a bigger loss when that block is removed.

**Supplementary Table 3 Permutation importance for amyloid and WMH blocks in Model 1c (B = 200).**

| Block permuted | Median $\boldsymbol{\Delta}$AICc [p25–p75] | Median $\boldsymbol{\Delta}\boldsymbol{R}_{\boldsymbol{marg}}^{\boldsymbol{2}}$ [p25–p75] | Median $\boldsymbol{\Delta}$logLik [p25–p75] |
| --- | --- | --- | --- |
| Amyloid block (permute PiB across visits within subject; recompute $\boldsymbol{PiB}^{\boldsymbol{2}}$ and interactions) | 671 [642–689] | 0.240 [0.224–0.252] | 335 [321–344] |
| WMH block (reassign WMH across persons at the ID level; keep WMH constant within person; recompute interactions) | 257 [228–270] | 0.143 [0.133–0.148] | 128 [114–135] |

Note: Larger positive medians indicate a greater drop in fit/explained variance when that block’s information is disrupted.

**Supplementary Table 4 Summary of Moderated mediation Models (Model 5b and Model 5c).**

|  | **MTC** | | | **Last CDR-SB** | | | **MTC** | | | **Last CDR-SB** | | |
| --- | --- | --- | --- | --- | --- | --- | --- | --- | --- | --- | --- | --- |
| *Predictors* | *Estimates* | *CI* | *p* | *Estimates* | *CI* | *p* | *Estimates* | *CI* | *p* | *Estimates* | *CI* | *p* |
| (Intercept) | 1.37 | 1.29 – 1.44 | **<0.001** | 0.57 | 0.17 – 0.97 | **0.005** | 1.21 | 1.16 – 1.27 | **<0.001** | 0.6 | 0.26 – 0.94 | **0.001** |
| Base CDR age (center at 65) | 0.00 | -0.00 – 0.01 | 0.155 | 0.02 | -0.00 – 0.04 | 0.111 | 0.00 | -0.00 – 0.00 | 0.808 | 0.01 | -0.01 – 0.03 | 0.210 |
| Female | -0.01 | -0.06 – 0.04 | 0.743 | -0.29 | -0.57 – -0.02 | **0.037** | 0.02 | -0.03 – 0.06 | 0.409 | -0.31 | -0.58 – -0.03 | **0.029** |
| College | -0.03 | -0.08 – 0.03 | 0.330 | -0.24 | -0.51 – 0.04 | 0.094 | -0.01 | -0.06 – 0.03 | 0.516 | -0.23 | -0.51 – 0.04 | 0.096 |
| Years between MTC and last CDR | -0.01 | -0.02 – 0.00 | 0.195 | 0.08 | 0.01 – 0.14 | **0.018** | -0.01 | -0.02 – 0.00 | 0.117 | 0.06 | -0.00 – 0.13 | 0.053 |
| WMH chronicity | 0.01 | 0.00 – 0.01 | **<0.001** | 0.02 | -0.00 – 0.05 | 0.050 |  |  |  |  |  |  |
| PiB chronicity | 0.01 | 0.01 – 0.01 | **<0.001** | 0.01 | -0.00 – 0.02 | 0.059 |  |  |  |  |  |  |
| WMH chronicity ^2 | 0.00 | -0.00 – 0.00 | 0.087 | 0.00 | 0.00 – 0.01 | **0.026** |  |  |  |  |  |  |
| WMH chronicity × PiB chronicity | 0.00 | 0.00 – 0.00 | **<0.001** | 0.00 | -0.00 – 0.00 | 0.865 |  |  |  |  |  |  |
| PiB chronicity × WMH chronicity^2 | 0.00 | -0.00 – 0.00 | 0.072 | 0.00 | -0.00 – 0.00 | 0.403 |  |  |  |  |  |  |
| Estimate WMH |  |  |  |  |  |  | 0.01 | 0.00 – 0.02 | **0.004** | 0.01 | -0.04 – 0.06 | 0.764 |
| Estimate Amyloid |  |  |  |  |  |  | 1.00 | 0.88 – 1.12 | **<0.001** | 0.62 | -0.32 – 1.56 | 0.194 |
| Estimate WMH ^2 |  |  |  |  |  |  | 0.00 | -0.00 – 0.00 | 0.055 | 0.00 | -0.00 – 0.00 | 0.175 |
| Estimate WMH × Amyloid |  |  |  |  |  |  | 0.12 | 0.08 – 0.15 | **<0.001** | 0.00 | -0.24 – 0.24 | 0.990 |
| Estimate Amyloid × WMH ^2 |  |  |  |  |  |  | 0.00 | -0.01 – -0.00 | **<0.001** | 0.00 | -0.01 – 0.01 | 0.906 |
| MTC |  |  |  | 1.74 | 1.14 – 2.35 | **<0.001** |  |  |  | 1.14 | 0.34 – 1.95 | **0.005** |
| MTC × PiB chronicity |  |  |  | 0.08 | 0.04 – 0.11 | **<0.001** |  |  |  |  |  |  |
| MTC × Estimate Amyloid |  |  |  |  |  |  |  |  |  | 2.96 | 1.38 – 4.54 | **<0.001** |
| Observations | 500 | | | 500 | | | 500 | | | 500 | | |
| R^2^ / R^2^ adjusted | 0.223 / 0.209 | | | 0.320 / 0.304 | | | 0.471 / 0.461 | | | 0.321 / 0.306 | | |

Regression estimates from moderated mediation models examining the influence of tau pathology on the relationship between amyloid and WMH burden and the last available CDR-SB score. The first two columns show results from the mediation model and the full moderated mediation model for Model 5b, which included WMH chronicity at the last CDR as the predictor, baseline amyloid chronicity as the moderator, and tau SUVR as the mediator. The last two columns present the mediation and full models for Model 5c, using estimated WMH at the last CDR and estimated baseline amyloid DVR values. Both models include covariates for sex, education, and the age difference between tau PET and the last CDR assessment. Bolded values indicate statistically significant predictors (p < 0.05).

**Supplementary Table 5 Summary of Moderated mediation Models (Model 6b and Model 6c).**

|  | **MTC** | | | **Annualized CDR change** | | | **MTC** | | | **Annualized CDR change** | | |
| --- | --- | --- | --- | --- | --- | --- | --- | --- | --- | --- | --- | --- |
| *Predictors* | *Estimates* | *CI* | *p* | *Estimates* | *CI* | *p* | *Estimates* | *CI* | *p* | *Estimates* | *CI* | *p* |
| (Intercept) | 1.178 | 1.118 – 1.238 | **<0.001** | 0.023 | 0.006 – 0.039 | **0.008** | 1.100 | 1.05 – 1.16 | **<0.001** | 0.030 | 0.01 – 0.04 | **0.001** |
| Base CDR age (center at 65) | 0.001 | -0.003 – 0.004 | 0.729 | 0.001 | -0.000 – 0.002 | 0.145 | 0.000 | -0.00 – 0.00 | 0.458 | 0.000 | -0.00 – 0.00 | 0.089 |
| Female | 0.010 | -0.037 – 0.056 | 0.678 | -0.007 | -0.019 – 0.006 | 0.306 | 0.020 | -0.03 – 0.06 | 0.444 | -0.010 | -0.02 – 0.01 | 0.262 |
| College | 0.003 | -0.044 – 0.050 | 0.899 | -0.005 | -0.018 – 0.008 | 0.483 | -0.010 | -0.06 – 0.03 | 0.568 | -0.010 | -0.02 – 0.01 | 0.401 |
| PiB chronicity at Tau | 0.022 | 0.019 – 0.024 | **<0.001** | -0.001 | -0.002 – 0.000 | 0.161 |  |  |  |  |  |  |
| WMH chronicity at Tau | 0.002 | -0.002 – 0.006 | 0.288 | 0.000 | -0.001 – 0.002 | 0.370 |  |  |  |  |  |  |
| PiB chronicity at Tau ^2 | 0.000 | 0.000 – 0.001 | **<0.001** | 0.000 | -0.000 – 0.000 | 0.831 |  |  |  |  |  |  |
| PiB chronicity at Tau × WMH chronicity at Tau | 0.001 | 0.001 – 0.001 | **<0.001** | 0.000 | -0.000 – 0.000 | 0.058 |  |  |  |  |  |  |
| WMH chronicity at Tau × PiB chronicity at Tau^2 | 0.000 | 0.000 – 0.000 | **<0.001** | 0.000 | -0.000 – 0.000 | 0.457 |  |  |  |  |  |  |
| Estimate Amyloid at Tau |  |  |  |  |  |  | 0.250 | 0.07 – 0.43 | **0.007** | -0.020 | -0.07 – 0.03 | 0.397 |
| Estimate WMH at Tau |  |  |  |  |  |  | 0.000 | -0.01 – 0.00 | 0.719 | 0.000 | 0.00 – 0.00 | **0.005** |
| Estimate Amyloid at Tau ^2 |  |  |  |  |  |  | 1.020 | 0.70 – 1.33 | **<0.001** | 0.010 | -0.08 – 0.11 | 0.819 |
| Estimate Amyloid at Tau × Estimate WMH at Tau |  |  |  |  |  |  | 0.010 | -0.00 – 0.03 | 0.140 | -0.010 | -0.02 – -0.00 | **0.001** |
| MTC |  |  |  | 0.169 | 0.141 – 0.196 | **<0.001** |  |  |  | 0.150 | 0.12 – 0.17 | **<0.001** |
| MTC × WMH chronicity at Tau |  |  |  | 0.010 | 0.006 – 0.014 | **<0.001** |  |  |  |  |  |  |
| MTC × Estimate WMH at Tau |  |  |  |  |  |  |  |  |  | 0.010 | 0.01 – 0.02 | **<0.001** |
| Observations | 460 | | | 460 | | | 460 |  |  | 460 |  |  |
| R^2^ / R^2^ adjusted | 0.406 / 0.396 | | | 0.367 / 0.353 | | | 0.443 / 0.435 |  |  | 0.350 / 0.337 |  |  |

Regression estimates from moderated mediation models examining the influence of tau pathology on the relationship between amyloid and WMH burden and the annualized CDR-SB score change. The first two columns show results from the mediation model and the full moderated mediation model for Model 6b, which included amyloid chronicity at tau as the predictor, WMH chronicity at tau as the moderator, and tau SUVR as the mediator. The last two columns present the mediation and full models for Model 6c, using estimated amyloid DVR at tau and estimated WMH values at tau. Both models include covariates for sex and education. Bolded values indicate statistically significant predictors (p < 0.05).

**Supplementary Table 6 Summary of Moderated mediation Models (Model 7b and Model 7c).**

|  | **MTC** | | | **Annualized CDR change** | | | **MTC** | | | **Annualized CDR change** | | |
| --- | --- | --- | --- | --- | --- | --- | --- | --- | --- | --- | --- | --- |
| *Predictors* | *Estimates* | *CI* | *p* | *Estimates* | *CI* | *p* | *Estimates* | *CI* | *p* | *Estimates* | *CI* | *p* |
| (Intercept) | 1.178 | 1.118 – 1.238 | **<0.001** | 0.020 | 0.003 – 0.036 | **0.023** | 1.100 | 1.05 – 1.16 | **<0.001** | 0.020 | 0.01 – 0.04 | **0.004** |
| c65base cdrage | 0.001 | -0.003 – 0.004 | 0.729 | 0.001 | -0.000 – 0.002 | 0.076 | 0.000 | -0.00 – 0.00 | 0.486 | 0.000 | 0.00 – 0.00 | **0.033** |
| Female | 0.010 | -0.037 – 0.056 | 0.678 | -0.007 | -0.020 – 0.006 | 0.286 | 0.020 | -0.03 – 0.06 | 0.443 | -0.010 | -0.02 – 0.00 | 0.197 |
| College | 0.003 | -0.044 – 0.050 | 0.899 | -0.006 | -0.019 – 0.007 | 0.391 | -0.010 | -0.06 – 0.03 | 0.575 | -0.010 | -0.02 – 0.01 | 0.265 |
| PiB chronicity at Tau | 0.022 | 0.019 – 0.024 | **<0.001** | 0.000 | -0.001 – 0.001 | 0.914 |  |  |  |  |  |  |
| WMH chronicity at Tau | 0.002 | -0.002 – 0.006 | 0.288 | 0.000 | -0.001 – 0.001 | 0.865 |  |  |  |  |  |  |
| PiB chronicity at Tau^2 | 0.000 | 0.000 – 0.001 | **<0.001** | 0.000 | -0.000 – 0.000 | 0.899 |  |  |  |  |  |  |
| PiB chronicity at Tau × WMH chronicity at Tau | 0.001 | 0.001 – 0.001 | **<0.001** | 0.000 | -0.000 – 0.000 | 0.595 |  |  |  |  |  |  |
| WMH chronicity at Tau × PiB chronicity at Tau^2 | 0.000 | 0.000 – 0.000 | **<0.001** | 0.000 | -0.000 – 0.000 | 0.250 |  |  |  |  |  |  |
| Estimate Amyloid at Tau |  |  |  |  |  |  | 0.250 | 0.07 – 0.44 | **0.007** | 0.030 | -0.03 – 0.08 | 0.392 |
| Estimate WMH at Tau |  |  |  |  |  |  | 0.000 | -0.01 – 0.00 | 0.652 | 0.000 | 0.00 – 0.00 | **0.040** |
| Estimate Amyloid at Tau^2 |  |  |  |  |  |  | 1.000 | 0.68 – 1.32 | **<0.001** | -0.100 | -0.22 – 0.01 | 0.068 |
| Estimate Amyloid at Tau× Estimate WMH at Tau |  |  |  |  |  |  | 0.010 | -0.03 – 0.04 | 0.703 | 0.010 | -0.00 – 0.02 | 0.122 |
| Estimate WMH at Tau × Estimate Amyloid at Tau ^2 |  |  |  |  |  |  | 0.010 | -0.05 – 0.07 | 0.720 | -0.020 | -0.04 – -0.00 | **0.025** |
| MTC |  |  |  | 0.102 | 0.067 – 0.137 | **<0.001** |  |  |  | 0.050 | 0.00 – 0.10 | **0.044** |
| MTC × PiB chronicity at Tau |  |  |  | 0.004 | 0.002 – 0.006 | **<0.001** |  |  |  |  |  |  |
| MTC × Estimate Amyloid |  |  |  |  |  |  |  |  |  | 0.220 | 0.13 – 0.30 | **<0.001** |
| Observations | 460 | | | 460 | | | 460 | | | 460 | | |
| R^2^ / R^2^ adjusted | 0.406 / 0.396 | | | 0.352 / 0.338 | | | 0.444 / 0.434 | | | 0.368 / 0.354 | | |

Regression estimates from moderated mediation models examining the influence of tau pathology on the relationship between amyloid and WMH burden and the annualized CDR-SB score change. The first two columns show results from the mediation model and the full moderated mediation model for Model 7b, which included WMH chronicity at tau as the predictor, amyloid chronicity at tau as the moderator, and tau SUVR as the mediator. The last two columns present the mediation and full models for Model 7c, using estimated WMH values at tau and estimated amyloid DVR at tau. Both models include covariates for sex and education. Bolded values indicate statistically significant predictors (p < 0.05).
